# Functional antibody and T-cell immunity following SARS-CoV-2 infection, including by variants of concern, in patients with cancer: the CAPTURE study

**DOI:** 10.21203/rs.3.rs-916427/v1

**Published:** 2021-09-20

**Authors:** Annika Fendler, Lewis Au, Scott T.C. Shepherd, Fiona Byrne, Maddalena Cerrone, Laura Amanda Boos, Karolina Rzeniewicz, William Gordon, Ben Shum, Camille L. Gerard, Barry Ward, Wenyi Xie, Andreas M. Schmitt, Nalinie Joharatnam-Hogan, Georgina H. Cornish, Martin Pule, Leila Mekkaoui, Kevin W. Ng, Eleanor Carlyle, Kim Edmonds, Lyra Del Rosario, Sarah Sarker, Karla Lingard, Mary Mangwende, Lucy Holt, Hamid Ahmod, Richard Stone, Camila Gomes, Helen R. Flynn, Ana Agua-Doce, Philip Hobson, Simon Caidan, Michael Howell, Mary Wu, Robert Goldstone, Margaret Crawford, Laura Cubitt, Harshil Patel, Mike Gavrielides, Emma Nye, Ambrosius P Snijders, James I MacRae, Jerome Nicod, Firza Gronthoud, Robyn L. Shea, Christina Messiou, David Cunningham, Ian Chau, Naureen Starling, Nicholas Turner, Liam Welsh, Nicholas van As, Robin L. Jones, Joanne Droney, Susana Banerjee, Kate C. Tatham, Shaman Jhanji, Mary O’Brien, Olivia Curtis, Kevin Harrington, Shreerang Bhide, Jessica Bazin, Anna Robinson, Clemency Stephenson, Tim Slattery, Yasir Khan, Zayd Tippu, Isla Leslie, Spyridon Gennatas, Alicia Okines, Alison Reid, Kate Young, Andrew J.S. Furness, Lisa Pickering, Sonia Gandhi, Steve Gamblin, Charles Swanton, Emma Nicholson, Sacheen Kumar, Nadia Yousaf, Katalin A. Wilkinson, Anthony Swerdlow, Ruth Harvey, George Kassiotis, James Larkin, Robert J. Wilkinson, Samra Turajlic

**Affiliations:** 1 Cancer Dynamics Laboratory, The Francis Crick Institute, London, NW1 1AT, UK; 2 Skin and Renal Units, The Royal Marsden NHS Foundation Trust, London, SW3 6JJ, UK; 3 Tuberculosis Laboratory, The Francis Crick Institute, London, NW1 1AT, UK; 4 Department of Infectious Disease, Imperial College London, W12 0NN, UK; 5 Retroviral Immunology Laboratory, The Francis Crick Institute, London, NW1 1AT, UK; 6 Research Department of Haematology at University College London Cancer Institute, WC1E 6DD, London, UK; 7 Autolus Limited, The MediaWorks, 191 Wood Lane, London, W12 7F; 8 Experimental Histopathology Laboratory, The Francis Crick Institute, London, NW1 1AT, UK; 9 Mass Spectrometry Proteomics Science Technology Platform, The Francis Crick Institute, London, NW1 1AT, UK; 10 Flow Cytometry Scientific Technology Platform, The Francis Crick Institute, London, NW1 1AT, UK; 11 Safety, Health & Sustainability, The Francis Crick Institute, London, NW1 1AT, UK; 12 High Throughput Screening Laboratory, The Francis Crick Institute, London, NW1 1AT, UK; 13 Advanced Sequencing Facility, The Francis Crick Institute, London, NW1 1AT, UK; 14 Department of Bioinformatics and Biostatistics, The Francis Crick Institute, London, UK; 15 Scientific Computing Scientific Technology Platform, The Francis Crick Institute, London, NW1 1AT, UK; 16 Metabolomics Scientific Technology Platform, The Francis Crick Institute, London, NW1 1AT, UK; 17 Department of Pathology, The Royal Marsden NHS Foundation Trust, London, NW1 1AT, UK; 18 Translational Cancer Biochemistry Laboratory, The Institute of Cancer Research, London, SW7 3RP, UK; 19 Department of Radiology, The Royal Marsden NHS Foundation Trust, London, SW3 6JJ, UK; 20 Gastrointestinal Unit, The Royal Marsden NHS Foundation Trust, London and Surrey SM2 5PT; 21 Breast Unit, The Royal Marsden NHS Foundation Trust, London, SW3 6JJ, UK; 22 Neuro-oncology Unit, The Royal Marsden NHS Foundation Trust, London, SW3 6JJ, UK; 23 Clinical Oncology Unit, The Royal Marsden NHS Foundation Trust, London, SW3 6JJ, UK; 24 Sarcoma Unit, The Royal Marsden NHS Foundation Trust and Institute of Cancer Research, London, SW3 6JJ, UK; 25 Palliative Medicine, The Royal Marsden NHS Foundation Trust, London, SW3 6JJ, UK; 26 Gynaecology Unit, The Royal Marsden NHS Foundation Trust, London, SW3 6JJ, UK; 27 Anaesthetics, Perioperative Medicine and Pain Department, The Royal Marsden NHS Foundation Trust, London, SW3 6JJ, UK; 28 Lung Unit, The Royal Marsden NHS Foundation Trust, London, SW3 6JJ, UK; 29 Head and Neck, The Royal Marsden NHS Foundation Trust, London, SW3 6JJ, UK; 30 Targeted Therapy Team, The Institute of Cancer Research, London, SW7 3RP, UK; 31 Haemato-oncology Unit, The Royal Marsden NHS Foundation Trust, London, SW3 6JJ, UK; 32 Acute Oncology Service, The Royal Marsden NHS Foundation Trust, London, SW3 6JJ, UK; 33 Department of Medical Oncology, 14th Floor, Great Maze Pond Road, Tower Wing, Guy’s Hospital, London SE1 9RY, UK; 34 Uro-oncology unit, The Royal Marsden NHS Foundation Trust, Surrey, SM2 5PT; 35 Neurodegeneration Biology Laboratory, The Francis Crick Institute, London, NW1 1AT, UK; 36 UCL Queen Square Institute of Neurology, Queen Square, London WC1N 3BG; 37 Structural Biology of Disease Processes Laboratory, The Francis Crick Institute, London, NW1 1AT, UK; 38 Cancer Evolution and Genome Instability Laboratory, The Francis Crick Institute, London, NW1 1AT, UK; 39 University College London Cancer Institute, London WC1E 6DD, UK; 40 Division of Genetics and Epidemiology and Division of Breast Cancer Research, The Institute of Cancer Research, London, SW7 3RP, UK; 41 Worldwide Influenza Centre, The Francis Crick Institute, London, NW1 1AT, UK; 42 Wellcome Center for Infectious Disease Research in Africa, University Cape Town, Observatory 7925, Republic of South Africa; 43 Equal contribution

**Keywords:** SARS-CoV-2, COVID-19, Cancer, Adaptive Immunity, Antibody Response, Neutralising Antibodies, T-cell Response, Prospective Study, Vaccine

## Abstract

Patients with cancer have higher COVID-19 morbidity and mortality. Here we present the prospective CAPTURE study (NCT03226886) integrating longitudinal immune profiling with clinical annotation. Of 357 patients with cancer, 118 were SARS-CoV-2-positive, 94 were symptomatic and 2 patients died of COVID-19. In this cohort, 83% patients had S1-reactive antibodies, 82% had neutralizing antibodies against WT, whereas neutralizing antibody titers (NAbT) against the Alpha, Beta, and Delta variants were substantially reduced. Whereas S1-reactive antibody levels decreased in 13% of patients, NAbT remained stable up to 329 days. Patients also had detectable SARS-CoV-2-specific T cells and CD4+ responses correlating with S1-reactive antibody levels, although patients with hematological malignancies had impaired immune responses that were disease and treatment-specific, but presented compensatory cellular responses, further supported by clinical. Overall, these findings advance the understanding of the nature and duration of immune response to SARS-CoV-2 in patients with cancer.

## Introduction

Patients with cancer have an increased risk of severe outcomes from coronavirus disease 2019 (COVID-19),^1,2^ with risk factors including general (e.g. increased age, male sex, obesity, comorbidities) as well as cancer-specific features (e.g. haematological and thoracic malignancies, active cancer, poor performance status).^3–8^ The precise effects of anti-cancer treatments on the course and outcome of SARS-CoV-2 infection are yet to be fully understood, with different reports yielding conflicting results.^5,7,9,10^ Understanding of the immune response to SARS-CoV-2 in this heterogeneous population, spanning multiple malignancy types and numerous treatment regimens, is crucial for optimal clinical management of those patients during the ongoing pandemic.

Calibration of current and future risk-mitigation measures, including risk of re-infection and vaccine effectiveness, requires an understanding of the impact of cancer and cancer treatments on the nature, extent and duration of immunity to SARS-CoV-2. Previous studies established an acute immune response to SARS-CoV-2 in cancer patients, with 1) solid tumour patients showing high seroconversion rates, and 2) haematological cancer patients showing impaired humoral immunity, especially under anti-CD20 treatments, but with improved survival in those with higher CD8+ T-cell counts.^11–13^ However, features of the immune response (including SARS-CoV-2-specific T-cells and neutralising antibodies), and their correlation with clinical characteristics in large non-hospitalized cancer cohorts, and cross-protection against emerging variants of concern (VOC) remain unknown.

CAPTURE (COVID-19 antiviral response in a pan-tumour immune monitoring study) is a prospective, longitudinal cohort study initiated in response to the global SARS-CoV-2 pandemic and its impact on cancer patients.^14^ The study evaluates the impact of cancer and cancer therapies on the immune response to SARS-CoV-2 infection and COVID-19 vaccinations. Here, we report findings from the SARS-CoV-2 infection cohort of the study.

## Results

### Patient demographics and baseline characteristics

**Figure 1:**
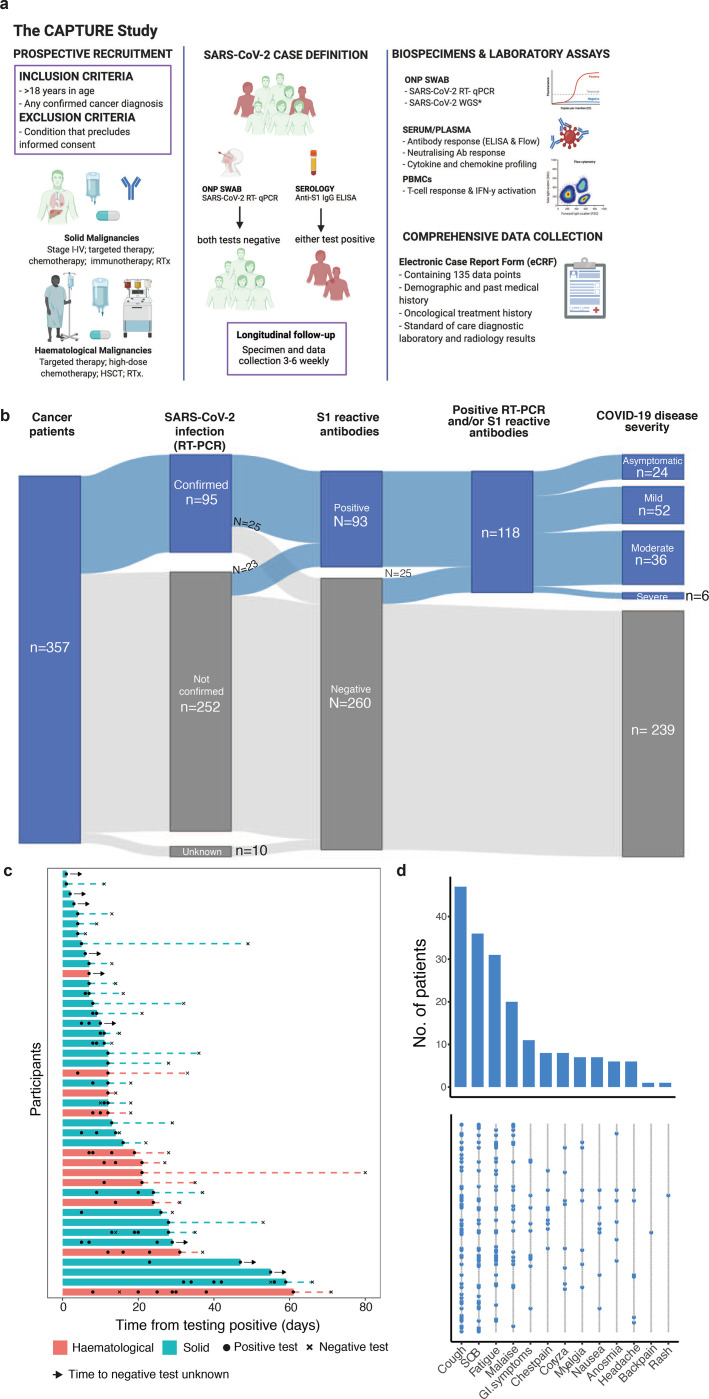
SARS-CoV-2 infection status, viral shedding, and COVID-19 symptoms of recruited patients. **a)** Patients with cancer irrespective of cancer type, stage, or treatment were recruited. Follow-up schedules for patients with cancer were bespoke to their COVID-19 status and account for their clinical schedules (inpatients: every 2 – 14 days; outpatients: every clinical visit maximum every 3–6 weeks in year one and every six months in year two, and at the start of every or every-second cycle of treatment). Clinical data, oronasopharyngeal swabs and blood were collected at each study visit. Viral antigen testing (RT-PCR on swabs), antibody (ELISA, flow cytometric assay), T cell response and IFN-γ activation assays were performed. **b)** Distribution of SARS-CoV-2 infection, and S1-reactive Ab status and COVID-19 severity in patients with cancer. 357 patients with cancer were recruited between May 4, 2020 and March 31st 2021. SARS-CoV-2 infection status by RT-PCR and S1-reactive Ab were analysed at recruitment and in serial samples. RT-PCR results prior to recruitment were extracted from electronic patient records. COVID-19 case definition includes all patients with either RT-PCR confirmed SARS-CoV-2 infection or S1-reactive Ab. **c)** Viral shedding in 43 patients with serial positive swabs. Solid bars indicate time to the last positive test, dotted lines denote the time from the last positive test to the first negative test. **d)** Distribution of symptoms in 118 COVID-19 patients. Bar graph denotes the number of patients. Each row in the lower graph denotes one patient. ONP, Oronasopharyngeal; ELISA, enzyme-linked immunoassay; PBMCs, peripheral blood mononuclear cells; WGS - whole genome sequencing, RTx, radiotherapy, HSCT, human stem cell transplant.

Between May 4, 2020 and March 31st 2021 (database lock), 357 unvaccinated cancer patients were evaluable and followed-up for a median of 154 days (IQR: 63–273). Median age was 59 years, 54% were male, 89% had solid malignancy, and the majority (64%) had advanced disease (Table 1). Overall, 118 patients (33%; 97 with solid cancers and 21 with haematological malignancies), were classified as SARS-CoV-2-positive according to our case definition (positive SARS-CoV-2 RT-PCR and/or ELISA for S1-reactive antibodies, at/or prior to study enrolment), and were included in the analysis (Figure 1a,b, see Methods). The most common comorbidities were hypertension (27%), obesity (21%) and diabetes mellitus (11%); no significant baseline differences were observed between patients with solid and haematological malignancies (Table 2, Supplementary Table 1). Overall, 88% patients received SACT (51% chemotherapy; 21% targeted therapy; 12% immune checkpoint inhibitors [CPI]; 5% anti-CD20), 10% had radiotherapy and 13% underwent surgery in the 12 weeks prior to infection. Response to the most recent anti-cancer intervention is shown in Table 2.

### Viral shedding and lineage

SARS-CoV-2 infection was confirmed by SARS-CoV-2 RT-PCR in 95/118 patients (81%). Repeat testing was not mandated by study protocol but 40% (47/118) had longitudinal swabs in the course of routine care. Within this group, the estimated median duration of viral shedding (see Methods) was 12 days (range: 6–80) (Figure 1c, Table 3), with evidence of prolonged shedding in patients with haematological malignancies (median 21 vs 12 days in patients with solid cancers) (Extended Data Figure 1a). Duration of viral shedding was not correlated with COVID-19 severity (r = 0.04, p = 0.7). We performed viral sequencing in 52 RT-PCR-positive samples with Ct > 32, of which 44/52 passed sequencing quality control. The Alpha VOC accounted for the majority of infections in our cohort between December 2020 and March 2021, consistent with community prevalence in the UK (Extended Data Figure 1b).

### Clinical correlates of COVID-19 severity in cancer patients

Overall, 94 patients (80%) were symptomatic, of whom 52 (44%) had mild, 36 (31%) moderate, and 6 (5%) severe illness (as per the WHO severity scale,^15^ Table 3); 24 patients (20%) were asymptomatic (WHO score 1). Among all patients (n=118), fever (47%), cough (42%), gastro-intestinal symptoms (12%), and dyspnoea (31%) were the most common presenting symptoms (Figure 1d), with a median of 2 symptoms reported (range: 0–7). In patients with a clear date of symptom resolution (n=77), duration of symptoms was 18 days (IQR: 11–30). Three patients met the criteria of long COVID (symptomatic > 90 days since presentation of disease (POD)), all following severe COVID-19 requiring ITU care.

Thirty-three patients (28%) were hospitalised due to COVID-19, with a median duration of in-patient stay of nine days (range: 1–120); 27 (23%) required supplemental oxygen, seven (6%) were admitted to an intensive care unit (ICU), with one (1%) requiring mechanical ventilation and inotropic support (Table 3). Thirteen patients (11%) were treated with corticosteroids (>10 mg prednisolone equivalent), and three patients (3%) received tocilizumab. Nine patients (8%) had a thrombo-embolic complication. At database lock, eleven SARS-CoV-2-positive patients (9%) died of progressive cancer, and two patients (2%) died due to recognised complications of COVID-19 (Table 3).

The risk of moderate and severe COVID-19 was associated with haematological malignancies, while risk of severe COVID-19 in solid malignancies was associated with progressive disease under SACT (Supplementary Table 2), in line with previous reports^7,8,12^. We found no association between COVID-19 severity, cancer stage, performance status, sex, age, obesity, smoking status or comorbidities across the whole cohort, though positive association of these factors were noted in registries largely reporting on cancer patients hospitalised with COVID-19^4,7,8,16,17^.

### Cytokine profiles and disease severity during infection

Due to the study design, recruitment was biased towards patients within the convalescent stage of infection. Twenty-seven patients (23%) were recruited while being RT-PCR-positive, and three (3%) became RT-PCR-positive after recruitment to CAPTURE. Cyto/chemokine profiling of 13 patients with acute infection (8 solid tumour, 6 haematological malignancy) indicated that IL-6, IL-8 IFN-y, IL-18, IL-9, IP-10, and MIP1-Beta levels were elevated compared to control (Extended Data Figure 1c,d, see Methods) and correlated with severe disease (Extended Data Figure 1e,f). Concentration of IFN-y and IL-18 in serum was significantly higher in patients with haematological malignancies^18^ (Extended Data Figure 1g).

### S1-reactive SARS-CoV-2 antibody response in cancer patients

**Figure 2:**
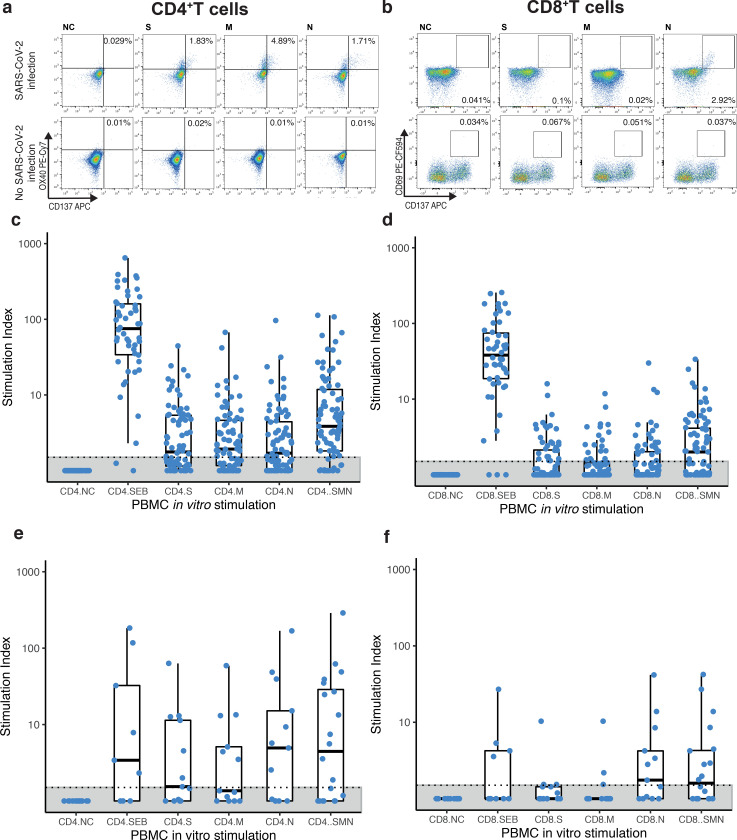
S1-reactive and antibody response in patients with cancer **a)** S1-reactive AbT by COVID-19 severity (n=112 patients). Significance was tested by Kruskal-Wallis test, p = 0.074. **b)** S1-reactive AbT by cancer type (Solid patients: n= 92, Haematological patients: n=20). Significance was tested by two-sided Wilcoxon Wilcoxon-Mann-Whitney U test, p = 0.011. **c)** NAbT by COVID-19 severity (n=112 patients). Significance was tested by Kruskal-Wallis test, p = 0.0027. **d)** NAbT by cancer type (Solid patient: n= 92, Haematological patients: n=20). Significance was tested by two-sided Wilcoxon-Mann-Whitney U test, p = 0.052. Boxes indicate 25 and 75 percentiles, line indicates median, and whiskers indicate 1.5 times the IQR. Dots represent individual samples. Dotted lines and grey boxes denote the limit of detection. **e)** Multivariate binary logistic regression evaluating association with lack of NAb in patients with cancer (n=112). Wald z-statistic was used two calculate two-sided p-values. *, p = 0.038. **f)** Multivariate binary logistic regression evaluating the association of lack of NAb in patients with solid cancer (n = 92). **g)** Multivariate binary logistic regression evaluating the association of lack of NAb in patients with solid cancer (n = 92). Dot denotes odds ratio (blue, positive odds ratio; red, negative odds ratio); whiskers indicate 1.5 times the IQR. **h)** NAbT against WT, Alpha, Beta, and Delta VOCs in patients (n=112) infected with WT SARS-CoV-2 or Alpha VOC. Violin plots denote density of data points. PointRange denotes median and 25 and 75 percentiles. Dots represent individual samples. Significance was tested by Kruskal Wallis test, p = 3.5e-07, two-sided Wilcoxon Mann Whitney U-test with Bonferroni correction (post-hoc test) was used for pairwise comparisons. p-values are denoted in the graph. **i)** S1-reactive AbT and **j)** NAbT post onset of disease (n=97 patients). Blue line denotes loess regression line with 95% confidence bands in grey. Black dots denote patients with one sample, coloured dots denote patients with serial samples (n=51 patients). Samples from individual patients are connected. Dotted lines and grey areas at bottom indicate limit of detection. NAb, neutralising antibody, NAbT, neutralising antibody titres, AbT, Antibody titres.

We evaluated total S1-reactive antibody titres by ELISA at multiple time-points throughout the study (with two median samples per patient [range: 1–10]). In total, 97/118 patients (82%) tested positive (85/95 [89%] solid tumours, 12/21 [57%] haematological malignancy); blood samples were not available for 2/118 patients (2%). Overall, 76/94 (81%) symptomatic and 21/24 (88%) asymptomatic patients had S1-reactive antibodies, and among the symptomatic patients there was a non-significant trend for higher S1-reactive antibody titres in those with higher COVID-19 severity (P = 0.057) (Figure 2a).

Thirteen patients (11%), with median follow up of 49 days (range: 14–344), had no evidence of S1-reactive antibodies but were positive by SARS-CoV-2 RT-PCR. Six further patients without detectable S1 antibody had no follow-up. Lack of seroconversion was significantly associated with haematological malignancies: 9/21 patients (43%) with haematological vs 10/97 patients (10%) with solid malignancies did not seroconvert (p = 0.0012). Antibody titres were also significantly lower in patients with haematological malignancies (Figure 2b). Two patients with long COVID did not seroconvert at any point during follow up.

A sensitive flow cytometric assay conducted on sera from a subset of patients with S1-reactive antibodies (n=40; Extended Data Figures 2a and 3), detected S-specific IgG in 38/40 (95%) (Extended Data Figure 2b) and IgM in 23/40 patients (58%) (Extended Data Figure 2c), with levels significantly correlated with S1-reactive antibody titres *(P <* 0.0001) (Extended Data Figure 2e-f). S-reactive IgA was detected in serum of only four patients (10%) (Extended Data Figure 2d), consistent with the role of IgA in early response to SARS-CoV-2 infection.^18^

Finally, we evaluated matched pre-pandemic sera from 47 patients, 10 with and 37 without S1-reactive antibodies in their sample collected during the pandemic. We found no evidence of S1-reactive antibodies in the pre-pandemic sera in any patient (Extended Data Figure 2g), but S-reactive IgG or IgM were detected in 18 patients without S1-reactive antibodies indicating cross-reactivity to seasonal human coronaviruses.

### NAbs against SARS-CoV-2 VOCs in cancer patients

We next performed a live virus neutralisation assay to evaluate whether patients’ sera could neutralise SARS-CoV-2 (see Methods). We measured either neutralising activity against wild-type (WT) SARS-CoV-2 or Alpha VOC, according to the causative variant (see Methods). We detected neutralising antibody (NAb) activity in 84/97 patients (87%) with S1-reactive antibodies (75/85 [88%] solid tumours, 8/12 [67%] haematological malignancy), with no significant differences in NAb titres (NAbT) by COVID-19 severity (Figure 2c). NAbT against WT were significantly lower in patients with haematological malignancies (Figure 2d). In a binary logistic regression model including all cancer patients (n=118), presence of haematological malignancy, but not comorbidities, age, sex, or COVID-19 severity was associated with lack of NAb (Figure 2e). In patients with solid tumours (n=97), there was no association with cancer type, stage, progressive disease or cancer therapy (Figure 2f,g). We were underpowered to evaluate patients with haematological malignancies (n=21), within a multivariate model.

In a subset of NAb-positive patients (N=34, 31 with solid malignancies, 3 with haematological malignancies; 25 with WT SARS-CoV-2 and 9 with Alpha VOC infection), we compared NAb against WT, Alpha, Beta, and Delta. In patients with WT infection, overall lower proportions of detectable responses (100% WT, 96% Alpha, 88% Beta, 85% Delta) were seen for VOC as well as lower NAbT vs the WT strain (Figure 2h). Considering patients with Alpha VOC infection, NAbT against Alpha VOC were increased vs WT and titres against Beta and Delta decreased vs. WT and Alpha.

There was a significant correlation between S1-reactive and NAbT for all variants (*P* < 0.01) (Extended Data Figure 2h); but we note that presence of S1-reactive antibodies was not always predictive of neutralising response, especially to VOCs.

### SARS-CoV-2 antibody response lasts up to 11 months

Next, we assessed antibody kinetics in 81/97 patients with S1-reactive antibodies and known timing of POD (n=70 solid tumours, n=11 haematological malignancy). We analysed a median of two timepoints per patient (range: 1–10) at a median follow-up of 56 days after POD (range: 1–344). Seventy-seven (95%) had S1-reactive antibodies at the time of enrolment (median 51 days after POD, range: 1–292, Figure 2f). Four patients (5%) had no antibodies at enrolment, but seroconverted between day 13–117 days POD. S1-reactive antibody titres showed a weak declining trend and 12 patients (15%) became seronegative 24–321 days POD: one T-ALL patient who following COVID-19 had a stem cell transplant complicated by chronic graft-versus-host disease, and 11 solid tumour patients with no unifying features to account for shorter-lived antibody response. Neutralising antibodies were detected as early as day one (Figure 2g), and as late as day 292 after POD and remained stable up to 329 days.

### SARS-CoV-2-specific T-cells are detected in cancer patients

**Figure 3:**
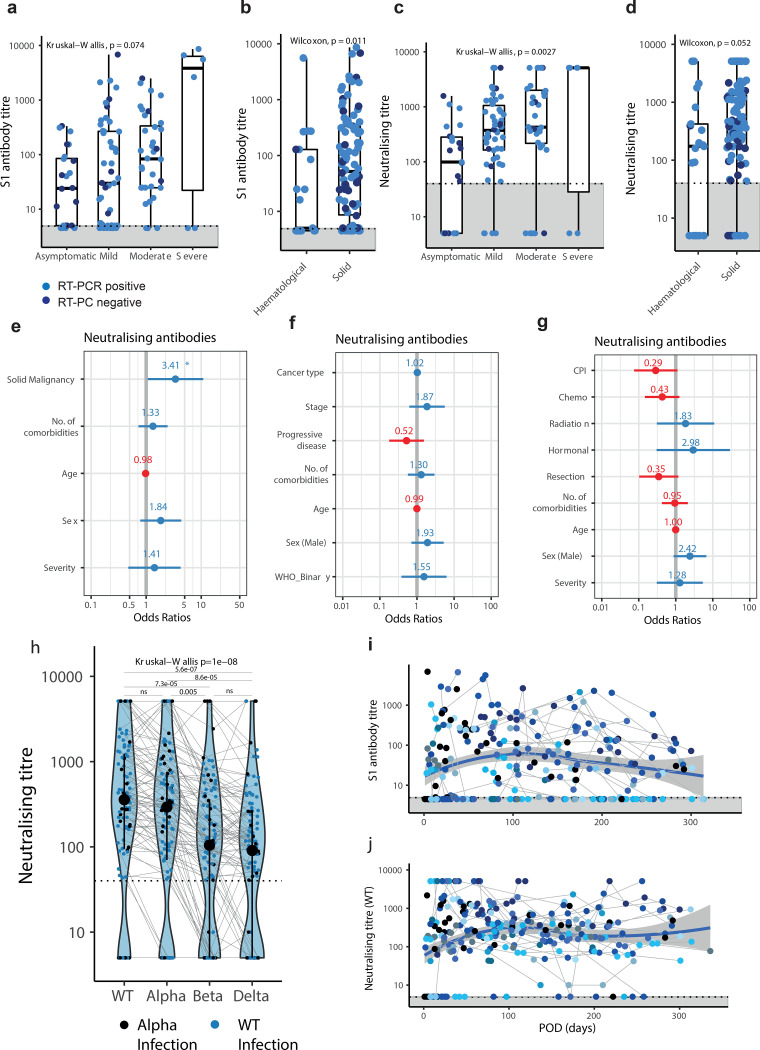
T cell response in patients with cancer **a,b)** Representative plots of CD4^+^CD137^+^OX40^+^ (CD4^+^) and CD8^+^CD137^+^CD69^+^ (CD8^+^) T cells in a patient with confirmed COVID-19 and a cancer patient without COVID-19 after in vitro stimulation with S, M, and N peptide pools, positive control (Staphylococcal enterotoxin B, SEB) or negative control (NC). Frequency of Sars-CoV-2-specific **c)** CD4^+^ and **d)** CD8^+^ T cells in solid patients with cancer (n= 83). Frequency of Sars-CoV-2-specific **e)** CD4^+^ and **f)** CD8^+^ T cells in haematological patients with cancer (n= 21). Stimulation index was calculated by dividing the percentage of positive cells in the stimulated sample by the percentage of positive cells in the negative control (NC). To obtain the total number of SsT cells the sum of cells activated by S, M, and N was calculated (SMN). Boxes indicate the 25 and 75 percentiles, line indicates the median, and whiskers indicate 1.5 times the IQR. Individual patients are represented as dots. Dots represent individual samples. Dotted lines and grey boxes denote the limit of detection. SsT cells, Sars-CoV-2-specific T cells.

PBMC stimulation assays (see Methods) were performed in 104/118 SARS-CoV-positive patients (n=83 solid tumour, n=21 haematological malignancy; Extended Data Figure 3b); 14 samples were excluded (for lack or low PBMC counts). SARS-CoV-2-specific CD4^+^ and CD8^+^ T-cells (SsT-cells; identified by activation induced markers OX40, CD137, and CD69)^19^ were measured (Figure 3a,b) at the first time point post-seroconversion (where evident), at the median of 54 days after POD (range: 1–292). We detected CD4^+^ T-cells in 79/104 (76%), and CD8^+^ T-cells and 54/104 patients (52%) (Figure 3c-f). CD4^+^ T-cells were detected in 81% of patients with solid malignancies, and in 41% of patients with haematological malignancies (Figure 3c,e). CD8+ T-cells were detected at similar frequencies (53% and 48%) across both malignancy types (Figure 3d,f) at a level consistently lower than CD4+ T-cells (Extended Data Figure 4a). The differences between CD8^+^ and CD4^+^ T-cell responses may be due to using 15-mer peptide pools for stimulation, though we note similar findings in non-cancer patients,^20,21,22^ indicating potential other factors, such as the broader range of antigens that induce CD8^+^ T-cells compared to CD4^+^ T-cells.^19^

Consistent with functional activation, IFN-*γ* secreted by SsT-cells,^23^ was detected after *in vitro* stimulation, and IFN-*γ* concentrations correlated with the number of SsT-cells (Extended Data Figure 4b).

Finally, as cross-reactive T-cell responses to HCoVs are observed frequently in healthy individuals,^19,24^ and given the lack of matched pre-infection samples in our cohort, we extended the T-cell assay to 12 cancer patients without confirmed SARS-CoV-2 infection. Cross-reactive CD4^+^ T-cells were detected in 7/12 and CD8^+^ T-cells in 3/12 participants, though the overall proportion of reactive T-cells was significantly lower than in patients with confirmed SARS-CoV-2 infection (*P*<0.05) (Extended Data Figure 4c,d).

### SsT-cell compensation in patients without humoral response

**Figure 4:**
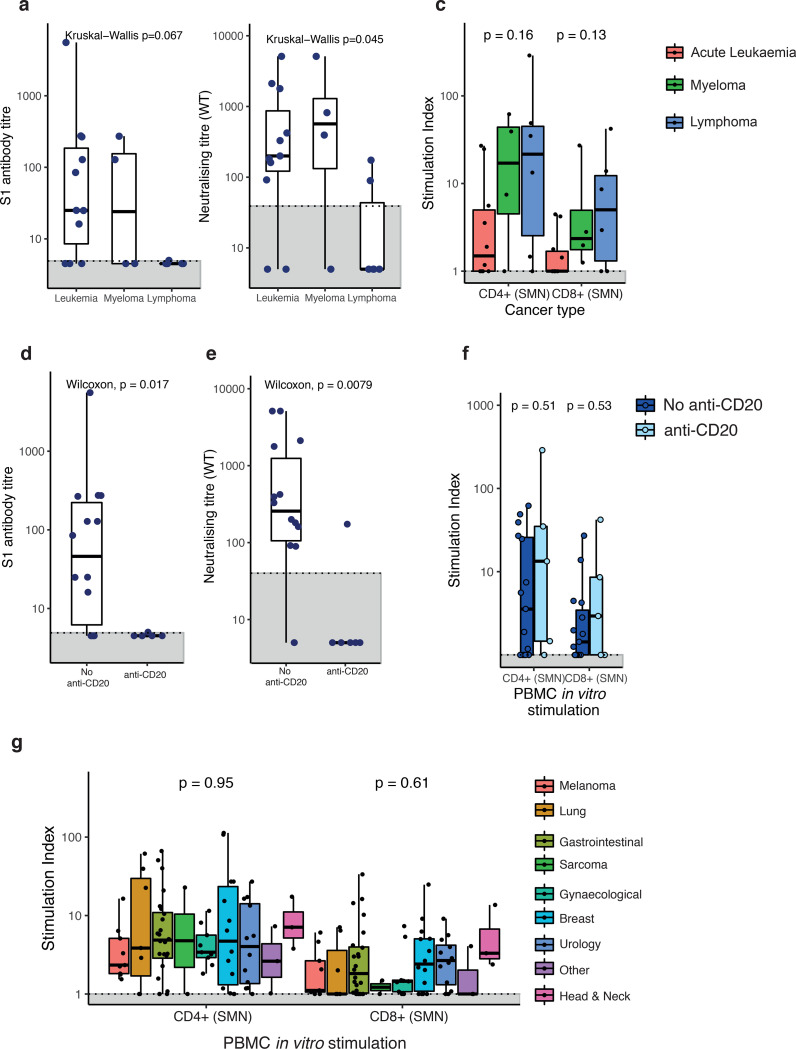
Comparison of antibody and T cell responses in patients with cancer **a)** S1-reactive AbT in patients with leukaemia (n=11), myeloma (n=4), and lymphoma (n=6). **b)** Neutralising antibody titres in patients with leukaemia (n=10), myeloma (n=4), and lymphoma (n=6). **c)** CD4^+^ and CD8^+^ cells T cells across patients with leukemia (n=10), myeloma (n=4), or lymphoma (n=6). Stimulation index was calculated by dividing the percentage of CD4^+^CD137^+^OX40^+^ (CD4^+^) and CD8^+^CD137^+^CD69^+^ (CD8^+^) T cells in the stimulated sample by the percentage of positive cells in the negative control (NC). Significance was tested by Kruskal-Wallis test, p < 0.05 was considered significant. **d)** S1-reactive AbT in patients with haematological malignancy receiving anti-CD20 treatment (n=6) vs other SACT (n=15). **e)** NAbT in patients with haematological malignancy receiving anti-CD20 treatment (n=6) vs other SACT (n=15). Significance was tested by two-sided Wilcoxon-Mann-Whitney U test, p < 0.05 was considered significant. **f)** Comparison of CD4^+^/CD8^+^ T cells between patients with haematological malignancies on anti-CD20 therapy (n=5, administered within six months) and not on anti-CD20 therapy (n=15). Significance was tested by two-sided Wilcoxon-Mann-Whitney U test, p < 0.05 was considered significant. **g)** CD4^+^ and CD8^+^ cells T cells across patients with solid cancer (n=81) by cancer subtype. Boxes indicate the 25 and 75 percentiles, line indicates the median, and whiskers indicate 1.5 times the IQR. Dots represent individual patient samples. Dotted lines and grey boxes denote the limit of detection. Significance was tested by Kruskal-Wallis test, p < 0.05 was considered significant. SACT, systemic anti-cancer therapy.

Patients with haematological malignancies had a wide range of antibodies (Figure 4a,b) and SsT-cell responses. In patients with leukaemia, NAb were detected in 6/11 and SsT-cells in 5/10 evaluable patients (two had both CD4+ and CD8+, two had CD4+ only, and one had CD8+ only). In patients with myeloma, 2/4 had NAb, and 3/4 had detectable SsT-cells (two both CD4+ and CD8+, one CD4+ only). None of the six lymphoma patients, including five who were treated with anti-CD20, had detectable NAbs, while SsT-cells were detected in 5/6 (three had both CD4+ and CD8+, one had CD4+ only, and one had CD8+ only). One further patient with AML treated with anti-CD20 had neither NAb nor SsT-cell responses. In total, we observed a discordance between antibody and T-cell responses amongst patients with haematological malignancy, whereby 7/9 patients with NAbT to WT SARS-CoV-2 lacked SsT-cell response (CD4+ and/or CD8+), and in 12 patients without NAb activity 7 had SsT-cell response. (Figure 4c,d, Supplementary Table 3). Overall, the levels of SsT-cells were higher in patients with lymphomas vs leukaemias (Figure 4e). The highest levels of SsT-cells was observed in a patient with diffuse large B-cell lymphoma and recent anti-CD20 therapy who had no detectable neutralising antibodies.

In patients with solid malignancies, the level of SsT-cells did not differ significantly by tumour type (Figure 4f) and the level of SARS-CoV-2-reactive CD4+ T-cells was positively correlated with S1-reactive antibody titres (Extended Data Figure 4e), which was not observed in patients with haematological malignancies (Extended Data Figure 4f). However, amongst 7/10 solid tumour patients without NAb response, 5 had detectable SsT-cells (3 both CD4+ and CD8+, 2 CD4+ only, Supplementary Table 2). Finally, following stimulation with S- and N- pools we observe that patients with haematological malignancy exhibit higher level of N-reactive compared to S-reactive CD8+ T-cells, (Figure 4e,f), while similar levels are observed in solid cancer patients (Figure 4c,d).

### T-cell responses are impacted in CPI-treated patients

**Figure 5:**
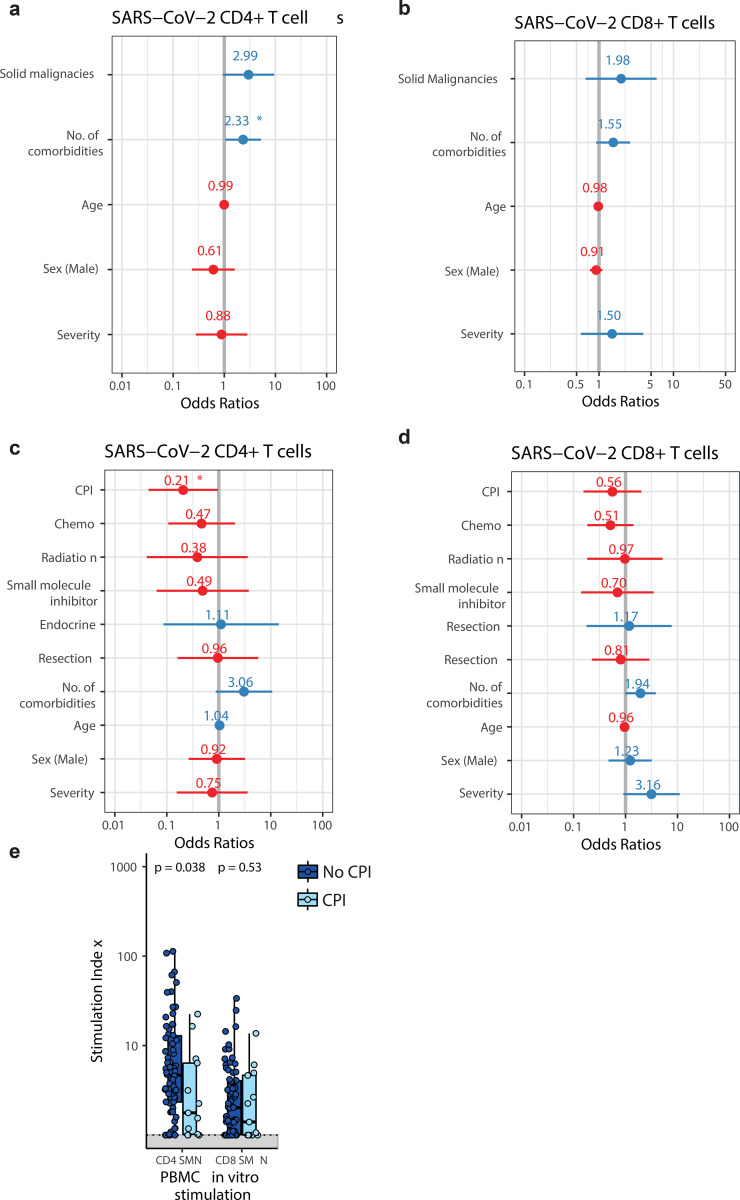
Associations between SARS-CoV-2-specific T cells with patient or cancer-specific features Multivariate binary logistic regression analysis evaluating associations between SARS-CoV-2-specific **a)** CD4^+^ and **b)** CD8^+^ T cells with cancer diagnosis (solid vs haematological malignancies), comorbidities, age, sex, and COVID-19 disease severity in 100 patients. Wald z-statistic was used two calculate two-sided p-values. *, p = 0.038. Multivariate binary logistic regression analysis evaluating associations between SARS-CoV-2-specific **c)** CD4^+^ and **d)** CD8^+^ T cells with anti-cancer intervention, age, sex, and COVID-19 disease severity in patients with solid cancer (n=81). Wald z-statistic was used two calculate two-sided p-values. *, p = 0.045. Dot denotes odds ratio (blue and red dots indicate positive or negative odds ratio, respectively); whiskers indicate 1.5 times the IQR. **e)** Comparison of SARS-CoV-2-specific CD4^+^/CD8^+^ T cells between patients with solid malignancies on CPI (n=13, administered within three months) and not on CPI (n=68). Boxes indicate the 25th and 75th percentiles, line indicates the median, and whiskers indicate 1.5 times the IQR. Dots represent individual samples. Significance was tested by two-sided Wilcoxon-Mann-Whitney U test (p = 0.038 and 0.53).

Next, we evaluated features associated with impaired T-cell responses to SARS-CoV-2 in cancer patients. We found no association between lack of SsT-cells with the presence of solid or haematological malignancies, nor with the number of comorbidities, age, sex, or COVID-19 severity (Figure 5a,b). In patients with solid malignancies, those on CPI (n=14) had significantly reduced levels of SARS-CoV-2 reactive CD4+ T-cells (Figure 5e), and in binary logistic regression model lack of SARS-CoV-2 reactive CD4+ (but not CD8+) T-cells was associated with CPI therapy within three months of SARS-CoV-2 infection (Figure 5c,d). Within the patients with haematological malignancies (n=21), anti-CD20 (n=4) was not associated with obvious reduction of SARS-CoV-2 reactive T-cells (Figure 5f).

## Discussion

Results from this prospective, longitudinal study of 118 SARS-CoV-2-positive cancer patients indicated that most patients with solid tumours developed a functional and durable (at least 11 months) humoral immune response to SARS-CoV-2 infection, as well as an anti-SARS-CoV-2-specific T-cell response. Patients with haematological malignancies had significantly lower seroconversion rates, and impaired immune responses that were both disease- and treatment-related (anti-CD20), although with evidence of compensation.

Most patients (82%) in our study had solid tumours and so findings largely reflect this cancer population. The majority (89%) of solid cancer patients seroconverted following SARS-CoV-2 infection (as evidenced by the presence of S1-reactive antibodies). Delayed/lack of seroconversion was observed in 10% of solid tumour patients, but no shared characteristics were identified among them. The observed high seroconversion rates in solid tumour patients were in line with data reported from smaller prospective studies conducted in the UK (95%, n=22)^12^ and Italy (88%, n=28);^25^ in both those studies seroconversion rates were similar to those observed in individuals without cancer. Recent studies in non-cancer subjects found a clear relationship between neutralising responses and vaccine efficacy.^26,27^ We now showed that 88% of seroconverted solid tumour patients also had functionally relevant NAb (against WT SARS-CoV-2 or Alpha, according to the causative variant). Importantly, whilst we observed a weak decline in S1-reactive antibody titres, NAbT were stable for up to 11 months of follow-up. In non-cancer population, inconsistent results have been reported regarding the length of persistence of both SARS-CoV-2-specific IgG and NAb over time,^20,28–31^ thus it is challenging to relate our data to those prior reports. In line with data for non-cancer SARS-CoV-2 convalescent patients^32^, we found that neutralising activity against Alpha, Beta, and Delta VOCs was decreased. This raises concerns about the ability of natural immunity to one variant to protect against other VOCs. Given the majority of cancer patients would now have been vaccinated against COVID-19, protection against evolving variants is critically relevant in the context of COVID-19 vaccine-induced immunity (companion paper Fender *et al*.)

SARS-CoV-2-infected cancer patients were previously shown to have depleted T-cells which showed markers of activation and exhaustion, and correlated with COVID-19 severity, but SsTcells were not evaluated.^12^ In our cohort, at a median of 54 days after POD, SsT-cells (including functional IFN-y expressing SsT-cells) were present in the majority of evaluated solid cancer patients (76%) and in half of the haematological malignancy patients (52%). Both in the acute and convalescent phase of SARS-CoV-2 infection, a significant proportion of SARS-CoV-2-specific CD4^+^ T-cells are T follicular helper cells (Tfh)^21,33^ which are required for IgG and neutralising response by B-cells.^34^ In our study, the number of CD4^+^ T-cells was significantly correlated with S1-reactive antibody titres in solid tumours, suggesting it may reflect Tfh T-cell activation and resulting B-cell activation. Overall, we found no variables associating with impaired T-cell responses to SARS-CoV-2 in cancer patients, except for CPI therapy within three months of SARS-CoV-2 infection (in solid tumours). It was previously shown that PD-1 blockade during acute viral infection can increase viral clearance by promoting CD8+ T-cell proliferation, but can also impair CD8+ T-cell memory differentiation, thereby impairing long-term immunity.^35^ While the role of PD-1 blockade on CD4+ T-cells during acute infection is less well understood, PD-1 signalling regulates expansion of CD4+ T-cells upon an immunogenic stimulus.^36^

We found an inverse relationship between antibody and SsT-cell responses in patients with haematological malignancies, whereby leukaemia patients had more pronounced antibody but impaired SsT-cell responses, while the opposite was observed for lymphoma patients. Generally, in patients with haematological malignancies immune responses were partially compensated, i.e. more robust SsT-cell responses, especially CD8+ T-cell responses, were detected in patients without antibody responses and vice versa. Furthermore, we found SsTcells in 4/5 evaluable patients on anti-CD20 treatment, of whom none had humoral responses. In total, all but one patient with haematological malignancies had mild or moderate disease, suggesting that SsT-cell responses, specifically CD8+ T-cells and non-spike-specific SsT-cells, can at least partially compensate for lacking humoral responses, although we note our cohort was largely convalescent. In one recent study, 10/13 patients with haematological malignancy and COVID-19 had SsT-cells, which were associated with improved survival (including in those on anti-CD20 therapy).^11^ Overall, the emerging data from our study and others^37^ appear to suggest that T-cell responses are likely important in those with haematological malignancies and may offer protection from severe COVID-19 in the absence of humoral responses.

The role of T-cells in protection from SARS-CoV-2 is not well understood, but T-cells were shown to play a crucial role in the clearance of acute SARS-CoV infection in mice.^38^ In line with this, early induction of functional SsT-cells was demonstrated to associate with rapid viral clearance and mild disease in COVID-19 patients,^39^ and preclinical animal studies suggest a role for cellular immunity in SARS-CoV-2 clearance.^40^ Importantly, SsTcells were shown to be induced by COVID-19 vaccines in both non-cancer^41,42^ and cancer (companion paper Fender *et al*.) population, and to have activity against VOCs.^43^ Furthermore, VOCs are not expected to escape SsTcell responses due to their highly multi-antigenic and multi-specific properties.^43^ In the general population, data indicate that SARS-CoV-2-specific memory T-cells are maintained beyond eight months following infection.^20,44^ In the context of the outbreak of SARS in 2003, SARS-specific T-cells were detected up to 17 years after infection, much longer than antibodies.^45^ An ongoing aim of the CAPTURE study is to evaluate the nature, durability, and clinical correlates of SsT-cell response in cancer patients as the pandemic evolves, especially in the context of COVID-19 vaccines.

This report has several limitations. Firstly, lack of a matched non-cancer cohort prevents direct comparisons between populations with and without cancer. Secondly, as mentioned above, the way patients are recruited into CAPTURE, including in the course of routine clinical care, may introduce selection bias, and thus our findings may not be fully generalizable to the wider cancer population. The fact that we recorded only two COVID-19-related deaths may be reflective of this (as well as the relatively low proportion of lung and haematological malignancies – the two cancer groups with increased COVID-19-related mortality).^3–6^ Furthermore, all but one patient with haematological malignancies in our cohort recovered, while 11/18 patients with blood cancers died due to COVID-19 at our institution^46^ before enrolment into CAPTURE commenced. Thus, it is possible that the patients with haematological malignancy in our analysis are not entirely representative of this population. Additional limitation pertains to our SsT-cell assessment - this was performed at a single time-point and so in instances where we did not detect a response, this might represent a timing bias rather than a lack of capacity to develop a response *per se*. As recruitment to CAPTURE commenced in May 2020 - which marked the end of the first wave of SARS-CoV-2 infections in the UK - most of the initially recruited participants were infected prior to study enrolment and evaluated in the convalescent phase. Even with the contribution of acutely infected patients recruited chiefly during the second wave, this analysis mainly assesses the immune protective response and its durability. Finally, some of the sub-group analyses are likely to be underpowered to robustly detect differences in immune response.

In summary, our data suggest that patients with solid malignancies are capable of developing humoral and cellular immunity against SARS-CoV-2, with NAb detectable for up to 11 months. In line with others,^11,12^ we found that patients with haematological malignancies had impaired humoral response, which was associated with malignancy type and anti-CD20 treatments, but was often linked to detectable SsT-cell responses. Finally, we found that neutralising activity against VOCs was reduced in samples from patients infected with WT SARS-CoV-2, which raises concerns about the effectiveness of naturally acquired immune responses against new SARS-CoV-2 VOCs. Whether such response can be boosted by COVID-19 vaccines remains under investigation in the vaccine cohort of CAPTURE, including the currently predominant Delta VOC (companion paper Fender *et al*.).

## Methods

### Study design

CAPTURE (NCT03226886) is a prospective, longitudinal cohort study that commenced recruitment in May 2020 at the Royal Marsden NHS Foundation Trust. The study design has been previously published.^14^ In brief, adult patients with current or history of invasive cancer are eligible for enrolment (Figure 1A). Inclusion criteria are intentionally broad, and patients are approached irrespective of cancer type, stage, or treatment. Patients with confirmed or suspected SARS-CoV-2 infection are targeted with broader recruitment in the course of routine clinical care (asymptomatic cases). Patients are screened at each study visit and classified as SARS-CoV-2-negative or SARS-CoV-2-positive based on a laboratory case definition of RT-PCR positive result and/or S1-reactive antibodies (details below). The primary endpoint is to describe the population characteristics of SARS-CoV-2 positive and negative cancer patients. The secondary endpoints include the impact of COVID-19 on long-term survival and ICU admission rates. Exploratory endpoints pertain to characterising clinical and immunological determinants of COVID-19 in cancer patients.

CAPTURE was approved as a substudy of TRACERx Renal (NCT03226886). TRACERx Renal was initially approved by the NRES Committee London, Fulham, on January 17, 2012. The TRACERx Renal substudy CAPTURE was submitted as part of Substantial Amendment 9 and approved by the Health Research Authority on April 30, 2020 and the NRES Committee London - Fulham on May 1, 2020. CAPTURE is being conducted in accordance with the ethical principles of the Declaration of Helsinki, Good Clinical Practice and applicable regulatory requirements. All patients provided written informed consent to participate.

### Study schedule and follow-up

Clinical data and sample collection for participating cancer patients is performed at baseline, and at clinical visits per standard-of-care management during the first year of follow-up; frequency varies depending on in- or outpatient status and systemic anti-cancer treatment regimens. For inpatients, study assessments are repeated every 2–14 days. For outpatients, the follow-up study assessments are aligned with clinically indicated hospital attendances. The frequency of study assessments in the first year for patients on anti-cancer therapies are as follows: every cycle for immune checkpoint inhibitors or targeted therapies; every second cycle for chemotherapy; every outpatient appointment (maximum 6 weekly) for patients on endocrine therapy or in surveillance or routine cancer care follow-up. Patient reported data is collected 3-monthly via an online questionnaire. In year two to five of follow-up, the frequency of study assessments is reduced (see **Supplementary Material Study Protocol**).

### Data and Sample Sources

Patient-reported outcome data are collected using PROFILES (Patient Reported Outcomes Following Initial treatment and Long-term evaluation of Survivorship; https://profiles-study.rmh.nhs.uk/). PROFILES is a web-based questionnaire administration and management system designed for the study of the physical and psychosocial impact of cancer and its treatment. Online questionnaires for baseline and follow up assessments were designed to record data for cancer patients participating in CAPTURE. Collected self-reported data include: ethnicity, smoking status, alcohol consumption, recent travel history, occupation, exercise habits, dietary habits, previous medical history, autoimmune disease (self, next of kin), vaccination history, concomitant medication, self-shielding status, previous SARS-CoV-2 tests, SARS-CoV-2 tests in household members, current and recent symptoms. Further demographic, epidemiological and clinical data (e.g. cancer type, cancer stage, treatment history) are collected from the internal electronic patient record system and entered into detailed case report forms in a secure electronic database. For information on anti-cancer intervention and response to most recent anti-cancer intervention, data was collected reflective of the time of SARS-CoV-2 infection per definition above where available, or the time of enrolment if data of disease onset is unknown (e.g. asymptomatic infections defined by positive serological positivity but negative/no RT-PCR results).

Study samples collected comprise blood samples, oropharyngeal swabs and archival and excess material from routine clinical investigations. Detailed sampling schedule and methodology has been previously described.^14^ Surplus serum from patient biochemistry samples taken as part of routine care were also retrieved and linked to the study IDs before anonymisation and study analysis. Collected data and study samples are de-identified and stored with only the study-specific study identification number. For self-reported data, a PROFILES member number is used, which is generated automatically.

### WHO classification of severity of COVID-19

We classified severity of COVID-19 according to the WHO clinical progression scale.^47^ Uninfected: uninfected, no viral RNA detected - 0; Asymptomatic: viral RNA and/or S1-reactive IgG detected – 1; mild (ambulatory): symptomatic, independent – 2; symptomatic, assistance needed - 3; moderate (hospitalised): no oxygen therapy (if hospitalised for isolation only, record status as for ambulatory patient) – 4; oxygen by mask or nasal prongs - 5; severe (hospitalised): oxygen by non-invasive ventilation or high flow – 6; intubation and mechanical ventilation, pO2/FiO2 ≥ 150 or SpO2/FiO2 ≥ 200 – 7; mechanical ventilation, pO2/FiO2 < 150 (SpO2/FiO2 < 200) or vasopressors – 8; mechanical ventilation, pO2/FiO2 < 150 and vasopressors, dialysis, or extracorporeal membrane oxygenation - 9; Dead - 10.

### Cell lines and viruses

SUP-T1 cells stably transfected with spike or control vectors were obtained from M.P., and L.M.i. Vero E6 cells were from the National Institute for Biological Standards and Control, UK. The SARS-CoV-2 isolate hCoV-19/England/02/2020 was obtained from the Respiratory Virus Unit, Public Health England, UK, and propagated in Vero E6 cells.

### Handling of oronasopharyngeal swabs, RNA isolation and RT-PCR

SARS-CoV-2 RT-PCR was performed from oronasopharyngeal (ONP) swabs using a diagnostics assay established at the Francis Crick Institute. The complete standard operating procedure is available on the Crick Covid-19 consortium website: https://www.crick.ac.uk/research/covid-19/covid19-consortium. ONP swabs were collected in VTM medium, frozen within 24 hrs after collection, and stored at −80°C until processing. ONP swabs were handled in a CL3 laboratory inside a biosafety cabinet using appropriate personal protective equipment and safety measures, which were in accordance with a risk assessment and standard operating procedure approved by the safety, health and sustainability committee at the Francis Crick Institute. In brief, 100 μl of swab vial content was inactivated in 5 M Guanidinium thiocyanate and RNA isolated using a completely automated kit-free, silica bead-based method.

PCR detection of SARS-CoV-2 was performed from 10 μl extracted RNA using two kits depending on the date of test. Up to 6^th^ December 2020, samples were tested in duplicate using Real-Time Fluorescent RT-PCR Kit for Detecting 2019-nCoV (BGI). Positive, negative, and extraction controls were included on each plate. Runs were regarded as valid when negative control Ct values were >37 and positive controls when Ct values were <37. Samples were only considered positive if Ct values in both replicates were <37. From 7^th^ December 2020, tests were performed using TaqPath COVID-19 CE-IVD RT-PCR Kit (Thermo Fisher), this time without replicate. Positive and negative controls were included on each plate and samples reported positive if 2 or 3 SARS-CoV-2 targets had Ct value <37 and the internal control Ct <32. With both kits, samples with non-exponential amplification were excluded from analysis.

### Viral Sequencing

All PCR-positive samples with ORF1ab Ct value < 32 were selected for viral sequencing, representing 52 samples from 32 patients. Sequencing was performed either on Illumina or on Oxford Nanopore Technologies instruments. Oxford Nanopore libraries were prepared following the ARTIC nCoV-2019 sequencing protocol v3 (LoCost) (protocols.iohttps://protocols.io/view/ncov-2019-sequencing-protocol-v3-locost-bh42j8ye) and then sequenced for 20 hours on a MinION flowcell on a GridION instrument. The ncov2019-artic-nf pipeline (version v1.1.1; https://github.com/connor-lab/ncov2019-artic-nf) written in the Nextflow domain specific language (version 20.10.0)^48^ was used to perform the QC, variant calling and consensus sequence generation for the samples. The full command used was “nextflow run ncov2019-artic-nf --nanopolish --prefix $PREFIX --basecalled_fastq fastq_pass/ --fast5_pass fast5_pass/ --sequencing_summary sequencing_summary.txt --schemeVersion V3 --minReadsPerBarcode 1 --minReadsArticGuppyPlex 1 -with-singularity articncov2019-nanopore.img -profile singularity,slurm -r v1.1.1”. Illumina libraries were prepared following the CoronaHiT protocol with minor modifications^49^, pooled and then sequenced at 100bp paired end on HiSeq 4000. The nf-core/viralrecon pipeline (version 1.1.0)^50^ was used to perform the QC, variant calling and consensus sequence generation for the samples. The full command used was “nextflow run nf-core/viralrecon --input samplesheet.csv --genome ‘MN908947.3’ --amplicon_bed nCoV-2019.artic.V3.bed --protocol ‘amplicon’ --callers ivar --skip_assembly --skip_markduplicates --skip_fastqc --skip_picard_metrics --save_align_intermeds -profile crick -r 1.1.0”. 44/52 passed quality control (>50% consensus sequence) and lineage was obtained using PANGOLIN (https://github.com/cov-lineages/pangolin). In the absence of sequencing data to confirm the causative SARS-CoV-2 variant, all patients tested with ThermoFisher TaqPath RT-PCR kit that reported S-dropout were considered to be infected with Alpha VOC.

### Viral shedding

Duration of viral shedding was estimated from research and opportunistic swabs and was defined as the time from first positive swab to the last positive swab (preceded by at least one negative swab).

### Handling of whole blood samples

All blood samples and isolated products were handled in a CL2 laboratory inside a biosafety cabinet using appropriate personal protective equipment and safety measures, which were in accordance with a risk assessment and standard operating procedure approved by the safety, health and sustainability committee of the Francis Crick Institute. For indicated experiments, serum or plasma samples were heat-inactivated at 56°C for 30 minutes prior to use after which they were used in a CL1 laboratory.

### Plasma and PBMC isolation

Whole blood was collected in EDTA tubes (VWR) and stored at 4°C until processing. All samples were processed within 24 hours. Time of blood draw, processing, and freezing was recorded for each sample. Prior to processing tubes were brought to room temperature (RT). PBMC and plasma were isolated by density-gradient centrifugation using pre-filled centrifugation tubes (pluriSelect). Up to 30 ml of undiluted blood was added on top of the sponge and centrifuged for 30 minutes at 1000g at RT. Plasma was carefully removed then centrifuged for 10 minutes at 4000g to remove debris, aliquoted and stored at −80°C. The cell layer was then collected and washed twice in PBS by centrifugation for 10 minutes at 300 x g at RT. PBMC were resuspended in Recovery cell culture freezing medium (Fisher Scientific) containing 10% DMSO, placed overnight in CoolCell freezing containers (Corning) at −80°C and then stored at −80°C.

### Serum isolation

Whole blood was collected in serum coagulation tubes (Vacuette CAT tubes, Greiner) for serum isolation and stored at 4°C until processing. All samples were processed within 24 hrs. Time of blood draw, processing, and freezing was recorded for each sample. Tubes were centrifuged for 10 minutes at 2000 x g at 4°C. Serum was separated from the clotted portion, aliquoted and stored at −80°C.

### S1-reactive IgG ELISA

Ninety-six-well MaxiSorp plates (Thermo Fisher Scientific) were coated overnight at 4°C with purified S1 protein in PBS (3 μg/ml per well in 50 μl) and blocked for 1 hour in blocking buffer (PBS, 5% milk, 0.05% Tween 20, and 0.01% sodium azide). Sera were diluted in blocking buffer (1:50). Fifty microliters of serum were added to the wells and incubated for 2 hours at RT. After washing four times with PBS-T (PBS, 0.05% Tween 20), plates were incubated with alkaline phosphatase-conjugated goat anti-human IgG (1:1000, Jackson ImmunoResearch) for 1 hour. Plates were developed by adding 50 μl alkaline phosphatase substrate (Sigma Aldrich) for 15–30 minutes after six washes with PBS-T. Optical densities were measured at 405 nm on a microplate reader (Tecan). CR3022 (Absolute Antibodies) was used as a positive control. The cut-off for a positive response was defined as the mean negative value multiplied by 0.35 times the mean positive value.

### Flow cytometry for spike-reactive IgG, IgM, and IgA

SUP-T1 cells were harvested, counted and spike-expressing and control SUP-T1 cells were mixed in a 1:1 ratio. The cell mix was transferred into V-bottom 96-well plates at 20,000 cells per well. Cells were incubated with heat-inactivated sera diluted 1:50 in PBS for 30 minutes, washed with FACS buffer (PBS, 5% BSA, 0.05% sodium azide), and stained with FITC anti-IgG (clone HP6017, Biolegend), APC anti-IgM (clone MHM-88, Biolegend) and PE anti-IgA (clone IS11–8E10, Miltenyi Biotech) for 30 minutes (all antibodies diluted 1:200 in FACS buffer). Cells were washed with FACS buffer and fixed for 20 minutes in 1% PFA in FACS buffer. Samples were run on a Bio-Rad Ze5 analyser running Bio-Rad Everest software v2.4 and analysed using FlowJo v10.7.1 (Tree Star Inc.) analysis software. Spike-expressing and control SUP-T1 cells were gated and mean fluorescence intensity (MFI) of both populations was measured. MFI in control SUP-T1 cells was subtracted from MFI in spike-expressing SUP-T1 cells, and resulting values were divided by MFI in control SUP-T1 cells to calculate the specific increase in MFI. Values >2 were considered positive.

### Neutralising antibody assay against SARS-CoV-2

Confluent monolayers of Vero E6 cells were incubated with SARS-CoV-2 WT or Alpha virus and two-fold serial dilutions of heat-treated serum or plasma samples starting at 1:40 for 4 hrs at 37°C, 5% CO_2_, in duplicates. The inoculum was then removed and cells were overlaid with viral growth medium. Cells were incubated at 37°C, 5% CO_2_. At 24 hours post-infection, cells were fixed in 4% paraformaldehyde and permeabilized with 0.2% Triton X-100/PBS. Virus plaques were visualized by immunostaining, as described previously for the neutralisation of influenza viruses using a rabbit polyclonal anti-NSP8 antibody used at 1:1000 dilution and anti-rabbit-HRP conjugated antibody at 1:1000 dilution and detected by action of HRP on a tetramethyl benzidine-based substrate. Virus plaques were quantified and ID_50_ was calculated.

### High-throughput live virus microneutralisation assay

High-throughput live virus microneutralisation assays were performed for a subset of 37 patients for WT SARS-CoV-2, Alpha, Beta or Delta. High-throughput live virus microneutralisation assays were performed as described previously.^51^ Briefly, Vero E6 cells (Institute Pasteur) or Vero E6 cells expressing ACE2 and TMPRSS2 (VAT-1) (Centre for Virus Research)^52^ at 90–100% confluency in 384-well format were first titrated with varying MOI of each SARS-CoV-2 variant and varying concentrations of a control monoclonal nanobody in order to normalise for possible replicative differences between variants and select conditions equivalent to wild-type virus. Following this calibration, cells were infected in the presence of serial dilutions of patient serum samples. After infection (24 hrs Vero E6 Pasteur, 16hrs VAT-1), cells were fixed with 4% final Formaldehyde, permeabilised with 0.2% TritonX-100, 3% BSA in PBS (v/v), and stained for SARS-CoV-2 N protein using Alexa488-labelled-CR3009 antibody produced in-house and cellular DNA using DAPI7. Whole-well imaging at 5x was carried out using an Opera Phenix (Perkin Elmer) and fluorescent areas and intensity calculated using the Phenix-associated software Harmony 9 (Perkin Elmer). Inhibition was estimated from the measured area of infected cells/total area occupied by all cells. The inhibitory profile of each serum sample was estimated by fitting a 4-parameter dose response curve executed in SciPy. Neutralising antibody titres are reported as the fold-dilution of serum required to inhibit 50% of viral replication (IC50), and are further annotated if they lie above the quantitative (complete inhibition) range, below the quantitative range but still within the qualitative range (i.e. partial inhibition is observed but a dose- response curve cannot be fit because it does not sufficiently span the IC50), or if they show no inhibition at all. IC_50_ values above the quantitative limit of detection of the assay (>25600) were recoded as 3000; IC_50_ values below the quantitative limit of the assay (< 40) but within the qualitative range were recoded as 39 and data below the qualitative range (i.e. no response observed) were recoded as 35.

### PBMC stimulation assay

PBMC for in vitro stimulation were thawed at 37 °C and resuspended in 10 ml of warm complete medium (RPMI, 5% human AB serum) containing 0.02% benzonase. Viable cells were counted and 1×10^6^ to 2×10^6^ cells were seeded in 200 μl complete medium per well of a 96-well plate. Cells were stimulated with 4 μl/well PepTivator SARS-CoV-2 spike (S), membrane (M), or nucleocapsid (N) pools (i.e., synthetic SARS-CoV-2 peptide pools, consisting of 15-mer sequences with 11 amino acid overlap covering the immunodominant parts of the S protein and the complete sequence of the N and membrane M proteins), representing 1μg/ml final concentration per peptide (Miltenyi Biotec, Surrey, UK). Staphylococcal enterotoxin B (Merck, UK) was used as a positive control at 0.5μg/ml final concentration, negative control was PBS containing DMSO at 0.002% final concentration. PBMC were cultured for 24 hrs at 37°C, 5% CO_2_.

### Activation-induced marker assay

PBMC supernatants were collected for cytokine analysis after stimulation for 24 hours. Cells were washed twice in warm PBMC. Dead cells were stained with 0.5 μl/well Zombie dye V500 for 15 minutes at RT in the dark, then washed once with PBS containing 2% FCS (FACS buffer). A surface staining mix was prepared per well, containing 2 μl/well of each antibody for surface staining (see **key resources table** for a full list of antibodies) in 50:50 brilliant stain buffer (BD) and FACS buffer. PBMC were stained with 50 μl surface staining mix per well for 30 minutes at RT in the dark. Cells were washed once in FACS buffer and fixed in 1% PFA in FACS buffer for 20 min, then washed once and resuspended in 200 μl PBS. All samples were acquired on a Bio-Rad Ze5 flow cytometer running Bio-Rad Everest software v2.4 and analysed using FlowJo v10.7.1 (Tree Star Inc.) analysis software. Compensation was performed with 20 μl antibody-stained anti-mouse Ig, k / negative control compensation particle set (BD Biosciences, UK). 1×10^6^ live CD19−/CD14− cells were acquired per sample. Gates were drawn relative to the unstimulated control for each donor. T-cell response is displayed as a stimulation index by dividing the percentage of AIM-positive cells by the percentage of cells in the negative control. If negative control was 0 the minimum value across the cohort was used. When S, M, and N stimulation were combined the sum of AIM-positive cells was divided by three times the percentage of positive cells in the negative control. A 1.5-fold increase in stimulation index is considered positive.

### IFN-y ELISA

IFN-y ELISA was performed using the human IFN-y DuoSet ELISA (R&D Systems) according to the manufacturer’s instructions. Briefly, 96-well plates were coated overnight with capture antibody, washed twice in wash buffer then blocked with reagent diluent for 2 hrs at RT. 100 μl of PBMC culture supernatants were added and incubated for 1 hr at RT and washed twice in wash buffer. 100 μl detection antibody diluted in reagent diluent was added per well and incubated for 2 hrs at RT. Plates were washed twice in wash buffer. 100 μl streptavidin-HRP dilution was added to the plates and incubated for 20 minutes in the dark at RT, plates were washed twice in wash buffer. The reaction was developed using 200 μl substrate solution for 20 minutes in the dark at RT then stopped with 50 μl stop solution. Optical density was measured at 450 nm on a multimode microplate reader (Berthold). Serial dilutions of standard were run on each plate. Concentrations were calculated by linear regression of standard concentrations ranging 0–600 pg/ml and normalized to the number of stimulated PBMC. The assay sensitivity was 5 pg/ml.

### Multiplex immune assay for cytokines and chemokines

The preconfigured multiplex Human Immune Monitoring 65-plex ProcartaPlex immunoassay kit (Invitrogen, Thermo Fisher Scientific, UK) was used to measure 65 protein targets in plasma on the Bio-Plex platform (Bio-Rad Laboratories, Hercules, CA, USA), using Luminex xMAP technology. Analytes measured included APRIL; BAFF; BLC; CD30; CD40L; ENA-78; Eotaxin; Eotaxin-2; Eotaxin-3; FGF-2; Fractalkine; G-CSF; GM-CSF; Gro-Alpha; HGF; IFN-Alpha; IFN-gamma; IL-10; IL-12p70; IL-13; IL-15; IL-16; IL-17A; IL-18; IL-1Alpha; IL-1Beta; IL-2; IL-20; IL-21; IL-22; IL-23; IL-27; IL-2R; IL-3; IL-31; IL-4; IL-5; IL-6; IL-7; IL-8; IL-9; IP-10; I-TAC; LIF; MCP-1; MCP-2; MCP-3; M-CSF; MDC; MIF; MIG; MIP-1Alpha; MIP-1Beta; MIP-3Alpha; MMP-1; NGF-Beta; SCF; SDF-1Alpha; TNF-Beta; TNF-Alpha; TNF-R2; TRAIL; TSLP; TWEAK; VEGF-A. All assays were conducted as per the manufacturer’s recommendation.

### Statistics & Reproducibility

No statistical method was used to predetermine sample size but as many patients with SARS-CoV-2 infection were recruited as possible including patients with no history of infection to identify patients in routine care with asymptomatic infection. The experiments were not randomised. The investigators were not blinded to allocation during experiments and outcome assessment.

Data and statistical analysis were done in FlowJo 10 and R v3.6.1 in R studio v1.2.1335. Gaussian distribution of baseline characteristics was tested by Kolmogorov-Smirnov test and differences in patient groups were compared using Chi-squared test, Mann-Whitney or Kruskal-Wallis tests as appropriate. Statistical methods for each experiment are provided in the figure legends. Gaussian distribution was tested by Kolmogorov-Smirnov test. Mann-Whitney, Wilcoxon, Kruskal-Wallis, Chi^2^, Fisher’s exact test, and Friedman tests were performed for statistical significance. A p-value <0.05 was considered significant. The ggplot2 package in R was used for data visualization and illustrative figures were created with BioRender.com. Data are usually plotted as single data points and box plots on a logarithmic scale. For boxplots, boxes represent upper and lower quartiles, line represents median, and whiskers IQR times 1.5. Notches represent confidence intervals of the median. For correlation matrix analysis, spearman rank correlation coefficients were calculated between all parameter pairs using the corrplot package in R without clustering. For pairwise correlation spearman rank correlation coefficients were calculated. Multivariate binary logistic regression analysis was performed using the glm function with the stats package in R.

### Reporting Summary

Further information on research design is available in the Nature Research Reporting Summary linked to this article.

### Data availability

All requests for raw and analysed data, and CAPTURE study protocol will be reviewed by the CAPTURE Trial Management Team, Skin and Renal Clinical Trials Unit, The Royal Marsden NHS Foundation Trust (CAPTURE@rmh.nhs.uk) to determine if the request is subject to confidentiality and data protection obligations. Materials used in this study will be made available upon request. There are restrictions to the availability based on limited quantities. Response to any request for data and/or materials will be given within a 28 day period. Data and materials that can be shared would then be released upon completion of a material transfer agreement.

### Code availability

No unpublished code was used in this study.

## Supplementary Material

Supplement 1

Supplement 2

Supplement 3

Supplement 4

Supplement 5

Supplement 6

Supplement 7

Supplement 8

Supplement 9

## Figures and Tables

**Table 1:** CAPTURE cohort overview

	Cohort	SARS-CoV-2 infection	No SARS-CoV2 Infection

Cohort Characteristics	n= 357	n= 118	n= 239

Age, years (median, range)	59 (18–87)	60 (18–87)	60 (26– 82)
Male, n (%)	192 (54)	64 (54)	128 (54)
**Cancer diagnosis, n (%)**			
Skin	79 (22)	10 (8)	69 (29)
Gastrointestinal	71 (20)	30 (25)	39 (16)
Urology	62 (17)	15 (12)	48 (20)
Lung	41 (11)	8 (7)	33 (14)
Haematological	39 (11)	21 (17)	17 (7)
Breast	31 (9)	16 (13)	16 (7)
Gynaecological	22 (6)	9 (7)	13 (5)
Sarcoma	12 (3)	4 (3)	8 (3)
Head & Neck	6 (2)	5 (4)	1 (0)
Other	4 (1)	4 (3)	0 (0)

**Cancer stage, n (%)**			
Stage I-II	20 (6)	7 (6)	13 (5)
Stage III	72 (20)	22 (18)	50 (22)
Stage IV	229 (64)	70 (58)	159 (67)
Haematological	39 (11)	21 (17)	17 (7)
**Days of Follow up, median (IQR)**	154 (63–273)	110 (58–274)	164 (63–274)

**Table 2.** Oncological and medical history of SARS-CoV-2 positive patients

	N=118

**Past medical history**	

HTN	31 (27)
PVD/IHD/CVD	9 (8)
Diabetes Mellitus	14 (11)
Obesity, BMI>30, n (%)	25 (21)
Inflammatory/Autoimmune	7 (6)
**Smoking status**	
Current smoker	36 (31)
Ex-smoker	51 (43)
Never smoked	12 (10)
Unknown	19 (16)

**Oncological history**	

**Solid tumours, n=97**	
Disease status (in respect to last treatment)	

**SACT, palliative, n=74**	
CR/PR	27 (28)
SD	24 (24)
PD	23 (24)
**SACT, neoadjuvant or radical CRT**	8 (8)
**Surgery ± adjuvant SACT**	15 (15)
**Treatment within 12 weeks**	
**Systemic therapy**	
Chemotherapy	43 (44)
Small molecule inhibitor	15 (15)
Anti-PD(L)1 ± anti-CTLA4	14 (14)
Endocrine therapy	7 (6)
No treatment	5 (4)
**Local therapy**	
Surgery	15 (13)
Radiotherapy	11 (10)

**Haematological malignancies, n=21**	

**Diagnosis**	
Acute leukaemia	11 (52)
Lymphoma	6 (29)
Myeloma	4 (19)
**Disease status**	
MRD/CR	5 (24)
Partial remission	7 (33)
SD	3 (14)
PD/relapse/untreated acute presentation	7 (33)
**Treatment within 12 weeks**	
Chemotherapy	17 (81)
Targeted therapy	10 (48)
Anti-CD20 therapy	6 (29)
CAR-T	1 (5)
**Haematologic stem cell transplant**	
Auto/Allograft pre-COVID-19	6 (29)
Auto/Allograft post-COVID-19	2 (9)

AS, active surveillance; BMI, body mass index; CAR-T, Chimeric antigen receptor T cell; CD-20, B-lymphocyte antigen; CR, complete response; CRT, chemoradiotherapy; CRP, C-reactive protein; CTLA-4, cytotoxic T-lymphocyte associated protein 4; DM, diabetes mellitus; GVHD, graft versus host disease; Hb, haemoglobin; HTN, hypertension; IHD, ischaemic heart disease; IQR, interquartile range; mAb, monoclonal antibody; MRD, minimal residual disease; NED, no evidence of disease; N0, neutrophil; PCR, polymerase chain reaction; PD progressive disease; PD(L)-1, program death (ligand)-1; Plt, platelet; PVD, peripheral vascular disease; SACT, systemic anti-cancer therapy; SD, stable disease; WBC, white cell blood count; WHO, world health organization

**Table 3:** Clinical characteristics of COVID-19 illness

COVID-19 characteristics	n (%)

**Viral shedding status**	
PCR positive, n (%)	95 (81)
Duration of PCR positivity, days median (range)	12 (6–80)

**WHO Severity Score**	
1, Asymptomatic	24 (20)
2–3, Mild	52 (44)
4–5, Moderate	36 (31)
>5, Severe	6 (5)
**Admission to hospital**	
Not hospitalised	54 (49)
Admitted with COVID-19- like illness	33 (29)
COVID-19 illness during hospitalisation	30 (25)
Duration of admission, days; median (range)	9 (1 – 120)
**Complications of COVID-19**	
Required supplemental oxygen	27 (23)
Pneumonia	29 (25)
Venous/arterial thromboembolism	9 (8)
Admission to ITU	7 (6)
Need for mechanical ventilation/NIV	4 (3)
**COVID-19 directed therapy**	
Corticosteroids	13 (11)
Anti-IL6 mAB	3 (3)

**Laboratory Investigations, median (IQR)**	
**Haematology**	
Hb, g/DL	110 (93 – 128)
WBC, ×10^6/L	5.7 (3.4 – 8.0)
N0, ×10^6/L	3.8 (2.1– 5.5)
Plt, ×10^6/L	213 (130 – 299)
**Biochemistry**	
Creatinine, umol/L	60 (53 – 71)
CRP, mg/L	59 (23 – 134)

**Clinical outcomes and impact**	
**Survival**	
Deceased, n (%)	13 (10)
Death within 30 days of PCR positivity	4 (3)
**Primary cause death:**	
Progressive Cancer	11 (9)
Complications of COVID-19	2 (2)

CRP, C-reactive protein; Hb, haemoglobin; IL-6, interleukin-6; IQR, interquartile range; mAb, monoclonal antibody; NIV, non-invasive ventilation; N0, neutrophil; PCR, polymerase chain reaction; Plt, platelet; WBC, white cell blood count; WHO, World Health Organization;
