# Supplementary figures and images for: Functional antibody and T-cell immunity following SARS-CoV-2 infection, including by variants of concern, in patients with cancer: the CAPTURE study
Source: Res Sq. 2021 Sep 20:rs.3.rs-916427. Preprint. [Version 1] doi: 10.21203/rs.3.rs-916427/v1 (PMC8475970; doi:10.21203/rs.3.rs-916427/v1)

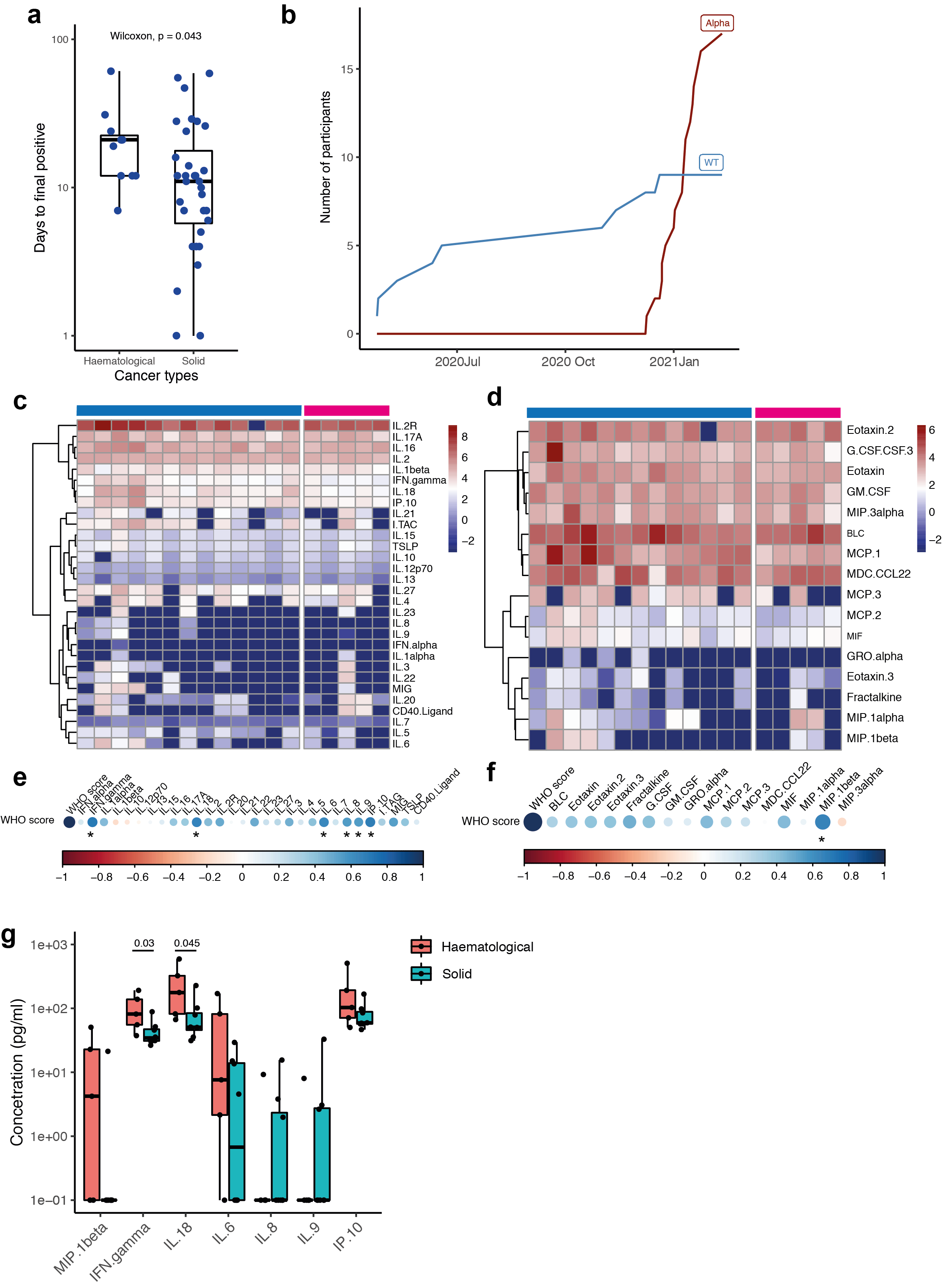

Supplement: Supplement 1 [file 6b4b5a1b84c9537cc0b31a3d.png]

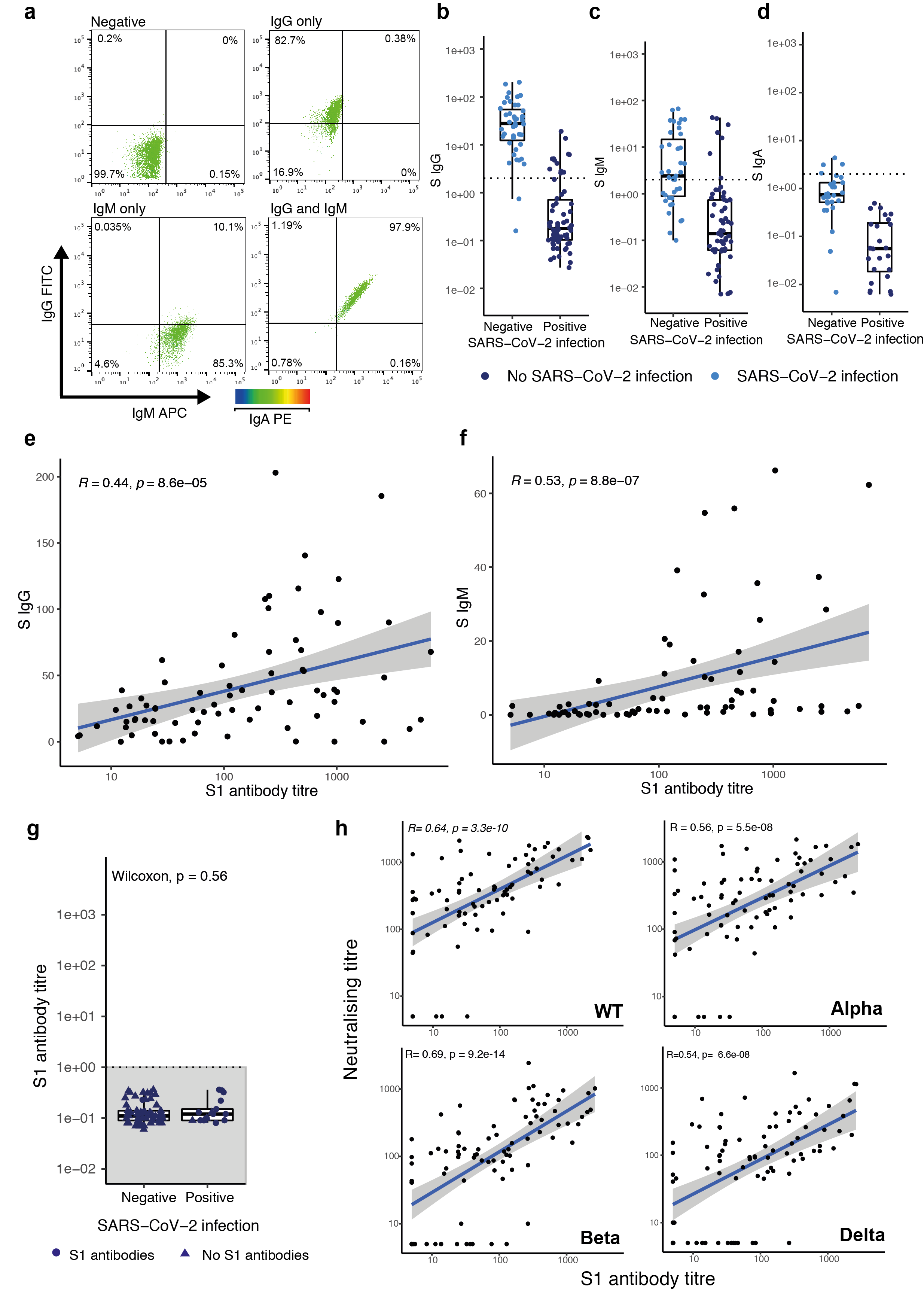

Supplement: Supplement 2 [file 9a55bc346ad9fc11e8a6fdbd.png]

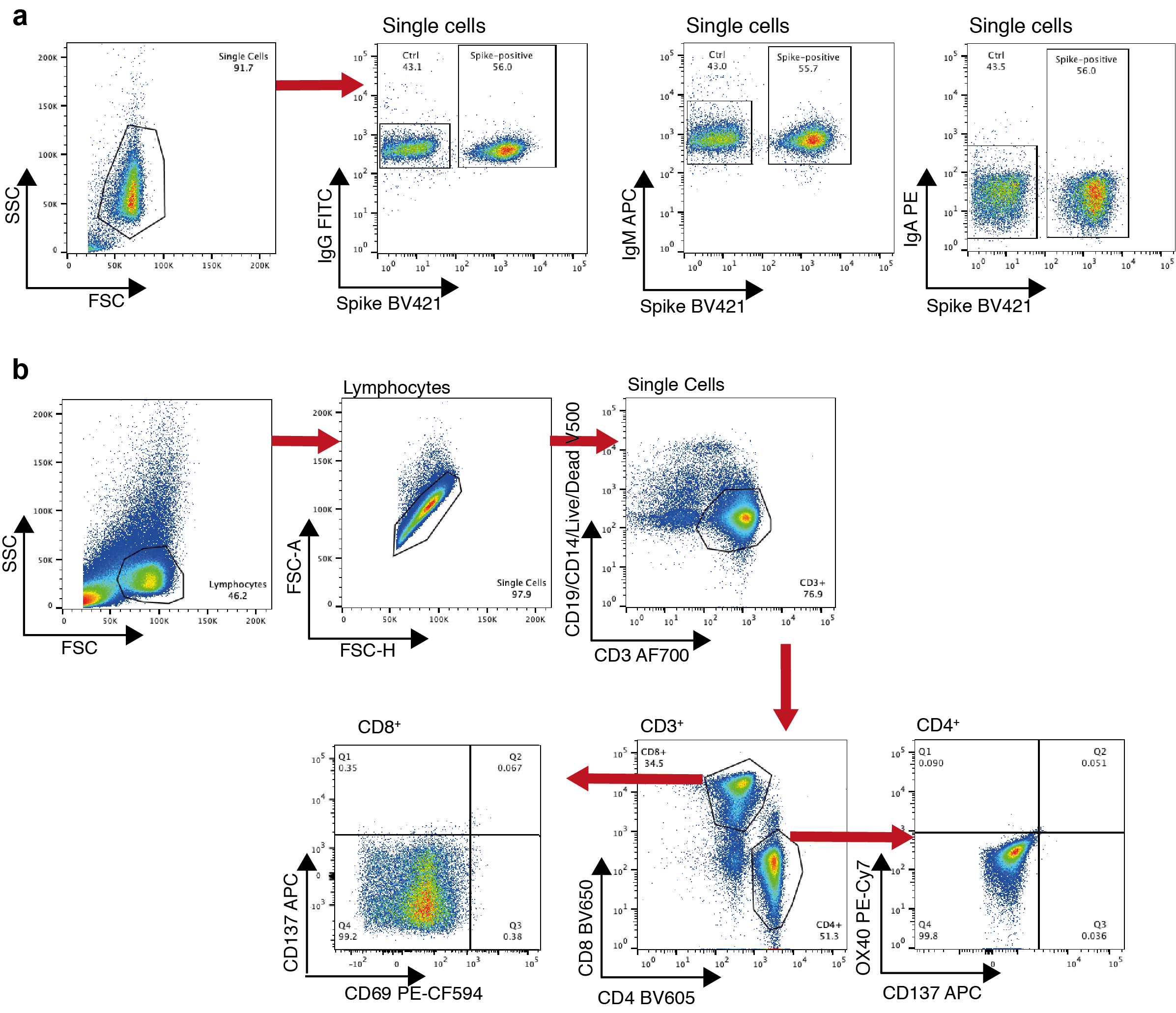

Supplement: Supplement 3 [file 5fb47c95ec77ef29de066d37.png]

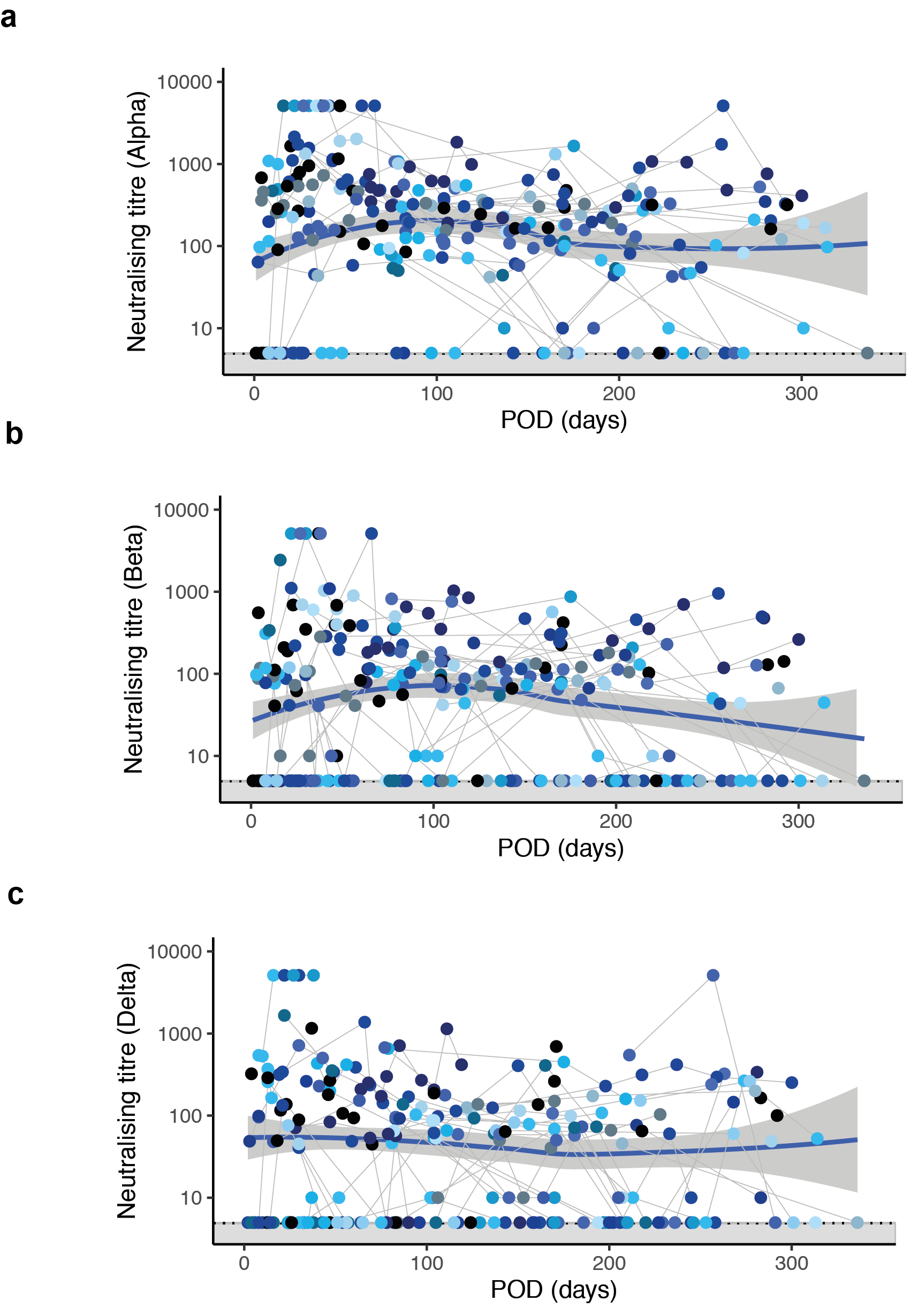

Supplement: Supplement 4 [file ec4170233efdc99df4ad169d.png]

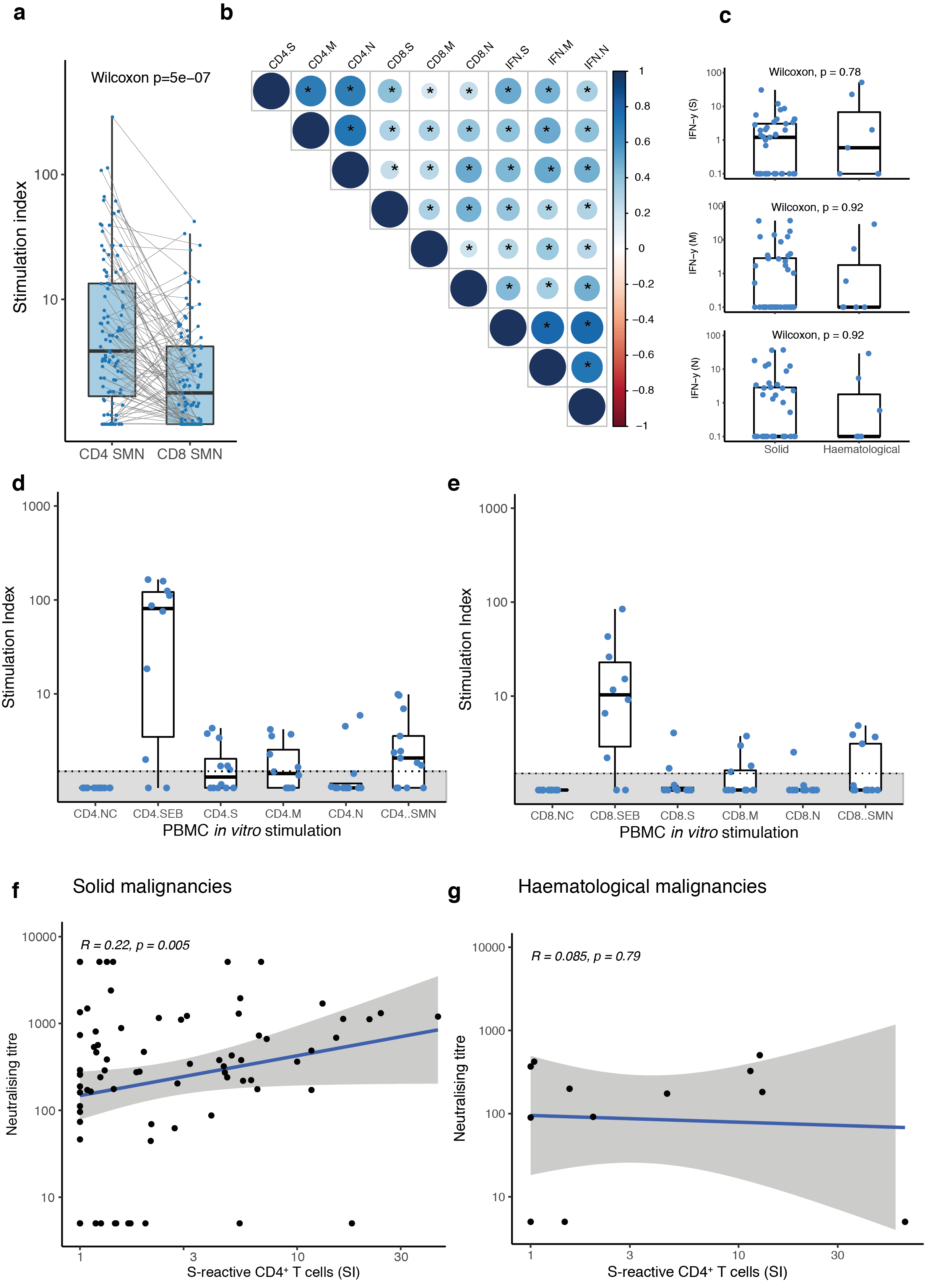

Supplement: Supplement 5 [file ac5a9c2d6d1d19e5e68a2569.png]

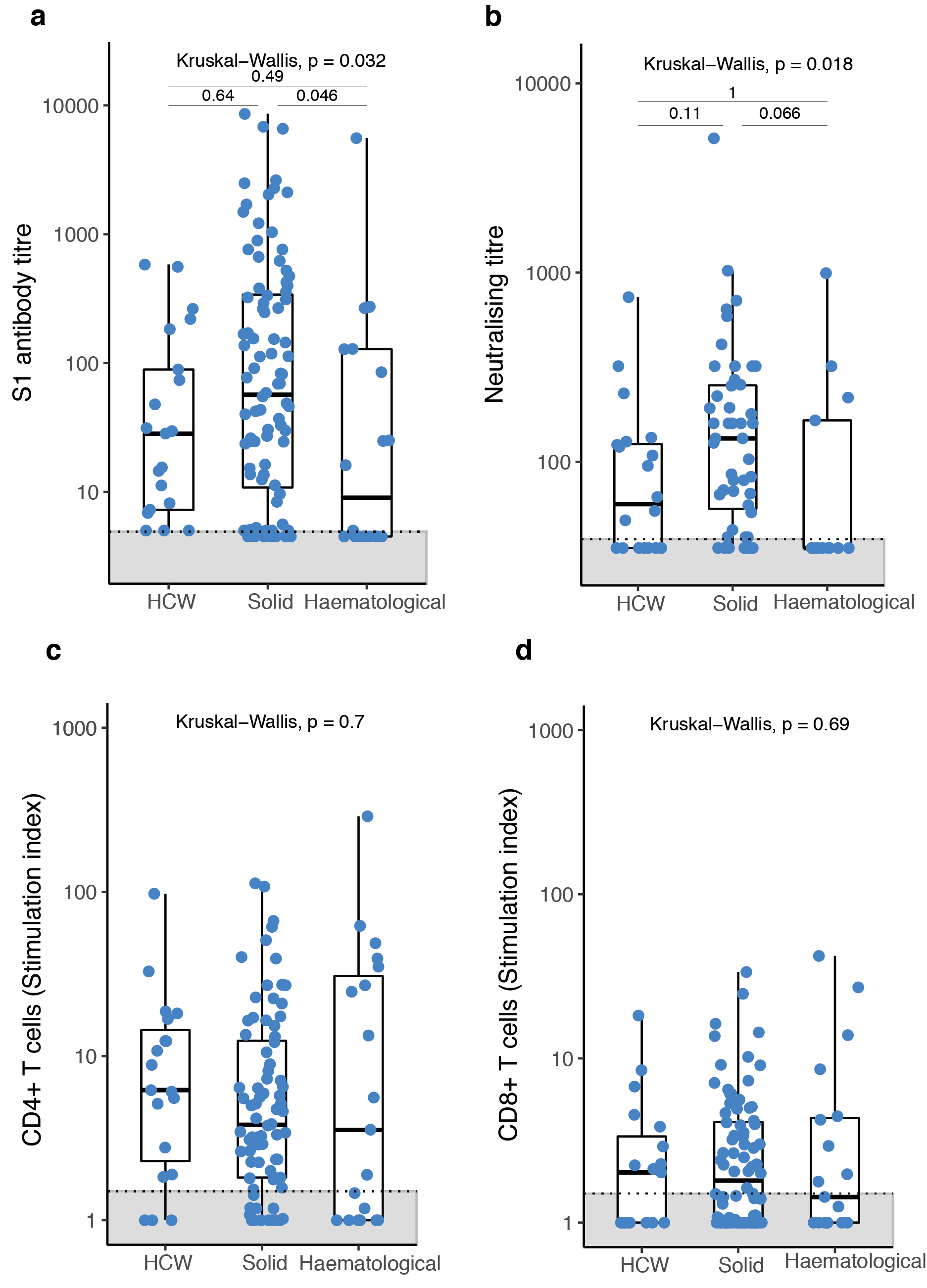

Supplement: Supplement 6 [file 0d815c40a5a2b38b95df6425.png]
