# Supplementary material for: Functional antibody and T-cell immunity following SARS-CoV-2 infection, including by variants of concern, in patients with cancer: the CAPTURE study
Source: Res Sq. 2021 Sep 20:rs.3.rs-916427. Preprint. [Version 1] doi: 10.21203/rs.3.rs-916427/v1 (PMC8475970; doi:10.21203/rs.3.rs-916427/v1)
Supplement: Supplement 7 [file ad5b9d2eeddbef9026231a28.pdf]

Extended Table 1: CAPTURE Cohort: comparison of haematological with solid tumours.

	Cohort	Solid Cancers	Haematological Malignancies	
Cohort Characteristics	n= 118	n= 97	n= 21	p-value*
Age, years (median, range)	60 (18-87)	61 (28-87)	58 (18- 85)	0.06
Male, n (%)	64 (54)	49 (51)	15 (71)	0.10
Past medical history				
HTN	31 (26)	26 (27)	5 (24)	0.77
PVD/IHD/CVD	9 (8)	6 (6)	3 (14)	0.20
Diabetes Melitus	14 (11)	11 (11)	3 (14)	0.71
Obesity, BMI>30, n (%)	25 (21)	22 (23)	4 (19)	0.71
Inflammatory/Autoimmune	7 (6)	6 (6)	1 (5)	0.80

*Mann Whitney or Chi-squared test, P<0.05 considered significant

BMI, body mass index; CVD, cerebrovascular disease; HTN, hypertension; IHD, ischaemic heart disease; PVD, peripheral vascular disease,
