# Supplementary material for: Functional antibody and T-cell immunity following SARS-CoV-2 infection, including by variants of concern, in patients with cancer: the CAPTURE study
Source: Res Sq. 2021 Sep 20:rs.3.rs-916427. Preprint. [Version 1] doi: 10.21203/rs.3.rs-916427/v1 (PMC8475970; doi:10.21203/rs.3.rs-916427/v1)
Supplement: Supplement 8 [file bf9d6557240f708cd5b60602.pdf]

Supplementary Table 2: Binary logistic regression

	COVID-19 Severity			COVID-19 Severity (severe only)		
Predictors	*Odds Ratios*	*CI*	*p*	*Odds Ratios*	*CI*	*p*
**Diagnosis**						
Haematological malignancy	3.09	1.02 – 9.26	**0.042***			
Skin	0.32	0.02 – 1.87	0.296			
Lung	2.14	0.41 – 9.57	0.325			
Gastrointerstinal	0.96	0.32 – 2.63	0.943			
Sarcoma	1.07	0.05 – 8.91	0.955			
Gynaecological	NA	NA	NA			
Breast	0.59	0.08 – 2.83	0.546			
Urology	1.18	0.30 – 3.93	0.797			
Other	1.12	0.05 – 9.38	0.922			
Head and Neck	NA	NA	NA			
solid cancers, cancer stage	2.39	0.81 – 10.46	0.166			
**Treatment**						
Checkpoint inhibitors	1.39	0.35 – 4.69	0.607			
Chemotherapy	0.63	0.24 – 1.54	0.317			
Radiotherapy	0.71	0.10 – 3.16	0.682			
Small Molecule Inhibitors	1.46	0.42 – 4.49	0.521			
Endocrine Therapy	3.3	0.57 – 17.08	0.156			
Surgery	0.22	0.01 – 1.21	0.16			
No treatment	0.22	0.01 – 1.21	0.16			
**Status**						
Solid cancers, advanced disease	1.94	0.69 – 6.35	0.234			
Solid cancers, progressive disease under SACT	1.57	0.57 – 4.16	0.373	17.18	2.38 – 353.26	**0.014**
**Demographics**						
Age	1.02	0.98 – 1.05	0.389			
Sex	1.4	0.58 – 3.53	0.462			
**Past medical history**						
ECOG	1.33	0.66 – 2.70	0.415			
Obesity	1.1	0.36 – 3.06	0.861			
Hypertension	1.14	0.41 – 3.40	0.805			
Vascular disease	0.28	0.07 – 0.98	0.052			
Diabetes mellitus	0.53	0.18 – 1.68	0.265			
Inflammatory/autoimmune disease	0.32	0.09 – 1.02	0.055			

NA, could not be fitted

Data are odds ratio and 97.5% CI in parentheses. Wald z-statistic was used two calculate two-tailed p-values.

Variables are adjusted for age and sex, age is adjusted for sex, sex is adjusted for age

ECOG=Eastern Cooperative Oncology Group.
