# Supplementary material for: Functional antibody and T-cell immunity following SARS-CoV-2 infection, including by variants of concern, in patients with cancer: the CAPTURE study
Source: Res Sq. 2021 Sep 20:rs.3.rs-916427. Preprint. [Version 1] doi: 10.21203/rs.3.rs-916427/v1 (PMC8475970; doi:10.21203/rs.3.rs-916427/v1)
Supplement: Supplement 9 [file 37137fd07f4328af9ac54683.pdf]

Supplementary Table 3: Neutralising antibody activity (NAb) to WT SARS-CoV-2 virus and presence or absence of SARS-CoV-2 specific T cells (SsT cells) in evaluable haematological patients (n = 19/20 evaluable) and patients with solid tumours, n=81/92 evaluable.

**Haematological malignancies**	**CD4+ SsT cells Present**	**CD4+ SsT cells Absent**	**CD8+ SsT cells Present**	**CD8+ SsT cells Absent**
**NAb +**	9 [69%]	4 [31%]	7 [54%]	6 [46%]
**NAb -**	2 [33%]	4 [67%]	2 [33%]	4 [67%]
**Solid malignancies**	**CD4+ SsT cells Present**	**CD4+ SsT cells Absent**	**CD8+ SsT cells Present**	**CD8+ SsT cells Absent**
**NAb +**	58 [84%]	11 [16%]	39 [57%]	30 [43%]
**NAb -**	8 [67%]	4 [33%]	2 [17%]	10 [83%]

NAb +, neutralising antibodies positive; NAb-, neutralising antibodies negative.
